# Speaking the host language: how *Salmonella* effector proteins manipulate the host

**DOI:** 10.1099/mic.0.001342

**Published:** 2023-06-06

**Authors:** Timesh D. Pillay, Sahampath U. Hettiarachchi, Jiyao Gan, Ines Diaz-Del-Olmo, Xiu-Jun Yu, Janina H. Muench, Teresa L.M. Thurston, Jaclyn S. Pearson

**Affiliations:** ^1^​ Centre for Bacterial Resistance Biology, Department of Infectious Disease, Imperial College, London SW7 2AZ, UK; ^2^​ The Francis Crick Institute, London NW1 1AT, UK; ^3^​ Centre for Innate Immunity and Infectious Diseases, Hudson Institute of Medical Research, Clayton, Victoria, Australia; ^4^​ Department of Microbiology, Monash University, Clayton, Victoria, Australia

**Keywords:** post-translational modification, *Salmonella *pathogenesis, type III effector protein, intracellular pathogen, biochemical mechanism

## Abstract

*
Salmonella* injects over 40 virulence factors, termed effectors, into host cells to subvert diverse host cellular processes. Of these 40 *Salmonella* effectors, at least 25 have been described as mediating eukaryotic-like, biochemical post-translational modifications (PTMs) of host proteins, altering the outcome of infection. The downstream changes mediated by an effector’s enzymatic activity range from highly specific to multifunctional, and altogether their combined action impacts the function of an impressive array of host cellular processes, including signal transduction, membrane trafficking, and both innate and adaptive immune responses. *Salmonella* and related Gram-negative pathogens have been a rich resource for the discovery of unique enzymatic activities, expanding our understanding of host signalling networks, bacterial pathogenesis as well as basic biochemistry. In this review, we provide an up-to-date assessment of host manipulation mediated by the *Salmonella* type III secretion system injectosome, exploring the cellular effects of diverse effector activities with a particular focus on PTMs and the implications for infection outcomes. We also highlight activities and functions of numerous effectors that remain poorly characterized.

## Introduction

*Salmonella enterica* is a major pathogen of humans and animals and the leading cause of bacterial foodborne illness globally [1–3]. The species can be broadly divided into human-restricted typhoidal or zoonotic non-typhoidal *Salmonella* (NTS) serovars. To date, over 2600 serovars of NTS have been characterized. Therefore, it is not surprising that they have a remarkably broad host range and diverse disease manifestations. Whereas typhoidal *S. enterica* typically cause invasive disease and typhoid fever in humans, NTS have multiple animal reservoirs and typically cause self-limiting gastroenteritis in humans. There is, however, an emerging global burden of invasive NTS (iNTS) infections, and as with typhoidal *Salmonella *, iNTS serovars are becoming increasingly multidrug-resistant [4–7].

*S. enterica* mediates much of its virulence through multiple genomic regions known as *Salmonella* pathogenicity islands (SPIs), two of which (SPI-1 and SPI-2) encode distinct functional type III secretion systems (T3SSs) [8] named accordingly as the SPI-1 and SPI-2 injectisomes. The *Salmonella* injectisomes have been most intensively studied in the prototype *S. enterica* serovar Typhimurium and collectively inject over 40 effector proteins, or effectors, with various functions (summarized in Table 1).

**Table 1.** SPI-1 and SPI-2 effector proteins and their functions Summary of SPI-1 and SPI-2 effector protein functions with reference to key interaction partners and biochemical activity. Interaction partners identified in high-throughput screens that have not been verified by a second method have been excluded. References are embedded for PDB structures only; other references can be found in the relevant section of the main text. nd, Not discovered.

Effector	Full name	SPI-1	SPI-2	PDB entry†	Biochemical activity	Interaction Partners	Functions	Related effectors‡
AvrA	Avirulence gene A	Y	Y	6BE0 [192]	Acetyltransferase	ERK2, MKK4*, MKK7*, p53	Inhibit inflammation and apoptosis	YopJ
CigR				4EW5 (C-term. domain) (unpublished)	Poorly defined		Anti-virulence effector inhibiting replication and SCV development	
GogB	Gifsy-one-gene B	Y	Y	–	Adaptor protein	SKP1, FBXO22	Inhibits NF-κB signalling	SspH1, SspH2, SlrP
GtgA (hom. PipA, GogA)	Gifsy-two-gene A	Y	Y	6GGR [223]	Zinc metalloprotease	Class II NF-κBs (p65, RelB and cRel)*	Inhibits NF-κB signalling	NleC
GtgE	Gifsy-two-gene E	Y	Y	5KDG [204] 5OED (C45A mut.) [206]	Cysteine protease	Rab32*, Rab29*, Rab38*	Prevents Rab accumulation on SCV and SITs	
PipB	Pathogenicity island-encoded proteins A-B2		Y	–	Poorly defined	PDZD8		PipB2
PipB2		Y	Y	2LEZ (NMR) (unpublished)	Poorly defined	Kinesin-1, KIF5B, annexin A2	Recruits kinesin-1 to the SCV to reorganize late endosome/lysosomes to promote bacterial survival	PipB
SifA	* Salmonella* induced filament proteins A and B	nd	Y	3CXB (w. SKIP) [183]	Adaptor protein	PLEKHM1, PLEKHM2, GDP-RhoA, Rab7, caspase-3	Induces SIT formation, detoxifies lysosomes, recruits late endosomes and lysosomes to SCV, maintains vacuolar membrane stability	SifB, SopE
SifB			Y	–	Poorly defined			SifA
SipA (SspA)	* Salmonella* invasion proteins A–D	Y		2FM9 [312]	Poorly defined	Caspase-3, F-actin, T-plastin, syntaxin8	Actin assembly, disruption of tight junctions, SCV positioning	
SipB		Y		3TUL (N-term) [313]	Translocation pore	Caspase-1	Pyroptosis	
SipC		Y		–	Translocation pore	Cytokeratin 8, cytokeratin 18, Exo70, F-actin, syntaxin 6	Actin nucleation, SCV maturation	
SipD			Y	3NZZ, 3O00 [314]			Bind bile salts ?bacterial sensor	
SlrP	* Salmonella* leucine-rich repeat protein	Y	Y	4PUF (w. thioredoxin) [83]	E3 ubiquitin ligase	Thioredoxin*, SNRPD2*, ERdj3, UbcH5b	Inhibits release of IL-1beta for apoptosis, antigen presentation of dendritic cells and inflammasome activation	SspH1, SspH2, IpaH family
SopA	* Salmonella* outer proteins A–F	Y		2QYU [315]	E3 ubiquitin ligase	TRIM56, TRIM65, UbcH5a, UbcH5c, UbcH7, HsRMA1, Caspase-3	Invasion, escape from SCV, PMN migration	NleL
SopB (SigD)		Y		4DID (w. Cdc42) [52]	Phosphoinositide phosphatase	Cdc42	Invasion, nuclear responses, SCV maturation, fluid secretion	IpgD
SopD		Y	Y	5CPC [243]	GAP and GEF	Rab8*, Rab10*	Invasion, inflammation, fluid secretion	
SopD2		nd	Y	5CQ9 [243]	GAP	Rab7*, Rab32*, AnxA2	Disrupt host-driven regulation of microtubule motors, impair trafficking of endocytic cargo to lysosomes for degradation	
SopE		Y		1GZS [255]	GEF	Cdc42*, Rac1*, Rab5*	Actin remodelling, phagosome–early endosome fusion, inflammation	
SopE2		Y		1R6E, 1R9K (NMR) [316]	GEF	Cdc42*, Rac1	Actin remodelling, inflammation	
SopF		Y	nd	7DN8 (w. ARF1), 7DN9 (w. NAD and ARF1) [170]	ADP ribosyltransferase	ATP6V0C*, ARF1	Preventing antibacterial autophagy (xenophagy)	
SptP	* Salmonella* protein tyrosine phosphatase	Y		1 G4W (w. Cdc42) [35] 1JYO (GAP domain w. chaperone) [317]	GAP and tyrosine phosphatase	Cdc42*, Rac1*, VCP*, vimentin*, cSrc*, NSF*, Syk*	Reversion of actin reorganization, inhibition of ERK activation	YopH, YopE, ExoS
SpvB	* Salmonella* plasmid virulence B–D	nd	Y	2GWJ-M [154]	ADP-ribosyl transferase	G-actin*	Inhibits F-actin polymerization, modifies actin to cause cytoskeletal disruption and apoptosis, promotes macrophage apoptosis and P-body disassembly	
SpvC		Y	Y	2 P1W (w. peptide), 2Q8Y (w. MAPK7), 2Z8M-P [42] 4H43 (H106N mut.), 4HAH [318]	Phosphthreonine lyase	ERK*, p38*, JNK*	Inhibit MAPK signalling, suppress pro-inflammatory in spleen and liver to facilitate bacterial growth	OspF, VirA, HopAI
SpvD		Y	Y	5LQ6 (R161 *var*.), 5LQ7 (G161 *var*.) [211]	Cysteine hydrolase	exportin-2*	Inhibits NF-kappaB signalling and interferes with host immune signalling to promote virulence	OspI, AvrPphB
SrfJ	SsrB regulated factor J	Y	Y	2WNW [319]	Putative glycoside hydrolase			
SseB	* Salmonella* secreted effectors B–L		Y	–	Undefined			
SseC			Y	–	Undefined			
SseD			Y	–	Undefined			
SseF		nd	Y	–	Transmembrane protein/adaptor	SseG, ACBD3, Rab1A, plakoglobin, desmoplakin, TIP60	Localizes, tethers and anchors SCV to Golgi network to facilitate bacterial replication; inhibits Rab1-mediated autophagy	SseG
SseG		nd	Y	–	Transmembrane protein/adaptor	SseF, ACBD3, Rab1A, plakoglobin, desmoplakin, Caprin-1	Localizes, tethers and anchors SCV to Golgi network to facilitate bacterial replication; inhibits Rab1-mediated autophagy	SseF
SseI (SrfH)			Y	4G2B (catalytic domain), 4 G29 (w. peptide) [215]	Cysteine deamidase	Gai2*, IQGAP1	Inhibits directional migration of macrophages and DCs, promoting systemic infection	SpvD, OspI, AvrPphB
SseJ			Y	–	Acyltransferase	GTP-RhoA, Cholesterol*	Cholesterol esterification to modify SCV membrane, reduce cellular cholesterol to inhibit SifA-cholesterol interaction and LAMP-1 vesicles	
SseK1		Y	Y	5H60 (w, UDP+Mn) [125]	Glycosyltransferase	FADD*, TRADD*, Rab1*, Rab5*, Rab11*, SseK1*	Inhibits TNF-alpha-stimulated NF-kappaB signalling and necroptosis	SseK2, SseK3, NleB effectors
SseK2		nd	Y	5H61, 5H62 (w. UDP), 5H63 (w. UDP-GlcNAc) [126]	Putative glycosyltransferase		Inhibits TNF-alpha-stimulated NF-kappaB signalling and necroptosis	SseK1, SseK3, NleB effectors
SseK3		nd	Y	6EYR, 6EYT (w. UDP-GlcNAc+Mn), 6CGI (w. UDP) [126]	Glycosyltransferase	TNFR1*, TRAILR*, SseK3*, TRIM32	Inhibits TNF-alpha-stimulated NF-kappaB signalling and necroptosis	SseK1, SseK2, NleB effectors
SseL		nd	Y	5HAF, 5UBW (catalytic domain) [108]	Deubiquitinase	K63 ubiquitin chains*, RPS3*, IkBα*, OSBP	Prevents accumulation of lipid droplets, inhibits autophagic clearance of cytosolic aggregates, induces late macrophage cell death	ChlaDub1, ChlaDub2, ElaD
SspH1	* Salmonella* secreted proteins H1–2	Y	Y	4NKH (LRR domain), 4NKG (LRR:PKN1 HR1b domain) [93]	E3 ubiquitin ligase	PKN1*, Ube2D	Ubiquitinates host kinase PKN1 for degradation, suppresses NF-kappaB activation, inhibits androgen steroid receptor and macrophage activation	SspH2, IpaH effectors
SspH2			Y	3 G06 [78]	E3 ubiquitin ligase	Nod1*, SGT1, UbcH5-Ubiquitin	Activates Nod1 signalling	SspH1, IpaH effectors
SteA	* Salmonella* translocated effectors A–E	Y	Y	–	Poorly defined	PI(4)P, Cullin-1	Regulates and partitions SCV vacuoles for bacterial growth	
SteB		Y	Y	–	Poorly defined			
SteC			Y	–	Kinase	MEK1*, HSP27*, FMNL1/2*	Induces formation of F-actin meshwork around SCV	
SteD			Y	–	Adaptor protein	mMHCII, TMEM127	Inhibits antigen presentation and T cell activation, suppresses adaptive immune responses, appears to act as an adaptor	
SteE		Y	Y	–	Adaptor protein	GSK3α/β, STAT3	Transcriptional reprogramming towards anti-inflammatory phenotype	

*Refers to interaction partners that are confirmed substrates.

†Protein database (PDB) files refer to structures obtained by crystallography unless otherwise stated.

‡Related effectors include those from *Salmonella* and other intracellular bacteria, listed here: AvrPphB from *Pseudomonas syringae*; *Chla*Dub1 and *Chla*Dub2 from *Chlamydia trachomatis*; ElaD from *Escherichia coli*; ExoS from *Pseudomonas aeruginosa*; HopAI from *Pseudomonas syringae*; IpaH effectors from *Shigella* spp*.* and enteroinvasive *E. coli* (EIEC); IpdG from *Shigella* spp*.*; NleB effectors from enteropathogenic *E. coli* (EPEC); NleC from *E. coli* and *Citrobacter rodentium*; NleL from *E. coli*; OspF from *Shigella flexneri*; OspI from *Shigella flexneri*; VirA from *Chromobacterium violaceum*; YopE is from *Yersinia* spp*.*; YopH from *Yersinia* spp*.*; YopJ family from *Yersinia enterocolitica.*

Remarkably, a single effector can have multiple host targets and/or functions, and even mediate ‘cross-talk’ within host cell signalling pathways during infection. While it is well established that SPI-1 effectors promote invasion of host epithelial cells [9], both SPI-1 and SPI-2 effectors contribute to the maturation and positioning of the unique intracellular membrane-bound niche of *Salmonella *, known as the *Salmonella* containing vacuole (SCV) [10]. Additionally, both SPI-1 and SPI-2 effector cohorts contribute to the subversion of innate immune responses and replication dynamics within the host [10–12].

The *Salmonella* effector repertoire is highly diverse in terms of biochemical functionality and is generally divided into enzymes and so-called adaptors that mediate no known direct enzymatic activity but often co-opt host enzymes. Those with enzymatic activity, like many Gram-negative pathogen effectors, mediate an impressive array of eukaryotic-like, biochemical post-translational modifications (PTMs) on host proteins, which in general promote infection [13]. PTMs represent a fundamental regulatory system that influences almost all aspects of normal host cell biology. PTMs including phosphorylation, ubiquitylation, glycosylation, nitrosylation, methylation, acetylation, lipidation and proteolysis can drastically affect the structural composition of host proteins, their subcellular localization, interactions and activities. They represent a highly effective mechanism by which bacteria can interfere with host cellular processes. As the ongoing discovery of host PTMs expands with the development of increasingly sensitive analytical techniques and deeper analysis of proteomic data [14, 15], the revelation of effector-mediated PTMs adds significantly to these processes.

**Fig. 1.**
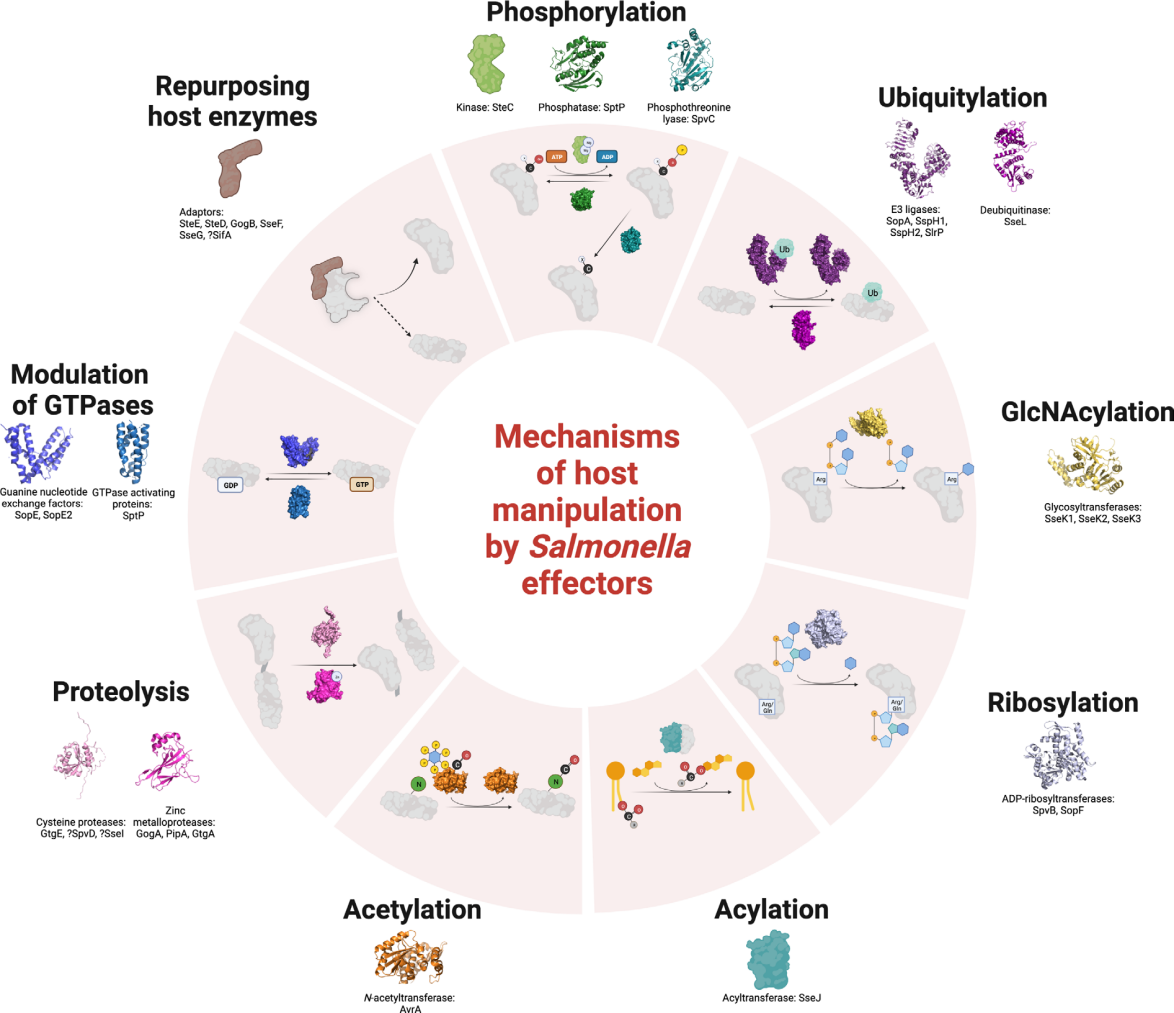
Summary of modifications mediated by *Salmonella* effectors. *Salmonella* effectors enact a range of host modifications, either enzymatically or through adaptor activity, to manipulate host cell processes, shown here in summary form. Effector proteins are categorized by colour corresponding to sections of the review, and demonstrated with their PDB structure in cartoon form (outside the circle) and surface form (in circle) using Pymol. Protein structures used here and in other figures were as follows: SptP (PDB: 1G4W, phosphatase and GAP domains used in corresponding parts of this figure, and phosphatase domain used in Fig. 2), SpvC (2P1W, here and Fig. 2), SopA (2QYU here and Fig. 3), SlrP (4PUF, Fig. 3), SspH2 (3G06, Fig. 3), SseL (5HAF, here and Fig. 3), SseK1 (5H60 here and Fig. 4), SseK2 (5HAF, Fig. 4), SseK3 (6EYR, Fig. 4), SpvB (2GWJ), AvrA (6BE0), GtgE (5KDG, here and Fig. 5), GtgA (6GGR) and SopE (1GZS). Where no relevant PDB structure is available, a generic protein symbol is used. A ‘?’ before the effector name refers to cases where the evidence of enzymatic activity is equivocal. Host substrates or co-factors are represented in grey. Symbols are either circular with atomic symbols (carbon [C], magnesium [Mg], nitrogen [N], oxygen [O] and zinc [Zn], with X referring to a variable atomic group), square signifying an amino acid by three-letter code (arginine [Arg]), skeleton chemical structures (UDP-GlcNAc in GlcNAcylation, glycerophospholipids in acylation, inositol hexakisphosphate in acetylation) and rectangles (ATP, ADP, GDP and GTP). The cartoon protein ‘Ub’ refers to ubiquitin. For a complete list of the *Salmonella* effector proteins see Table 1. Image created with BioRender.com.

Our understanding of effector-mediated PTMs has transformed the field and emphasized the complexity of *Salmonella* virulence mechanisms. In this review, we focus on the diverse ways in which *Salmonella* effectors use PTMs to manipulate eukaryotic signalling pathways, as summarized in Fig. 1. After more than 25 years of research since the discovery of the SPI-1 [16] and SPI-2 [17] secretion systems, our understanding of *Salmonella* effector functions represents one of the best characterized bacterial pathogens. Here we have grouped effectors according to the PTM they mediate, either directly or indirectly, and how this changes the cell status. Considering each of these examples, we highlight the diverse aspects of effector biology including the role of molecular mimicry, PTMs of effectors by host proteins or the effector itself, irreversible modifications, and the cross talk between different PTMs as well as co-operative effector functions. Finally, we review some of the less clearly defined activities and functions of poorly characterized effectors.

**Fig. 2.**
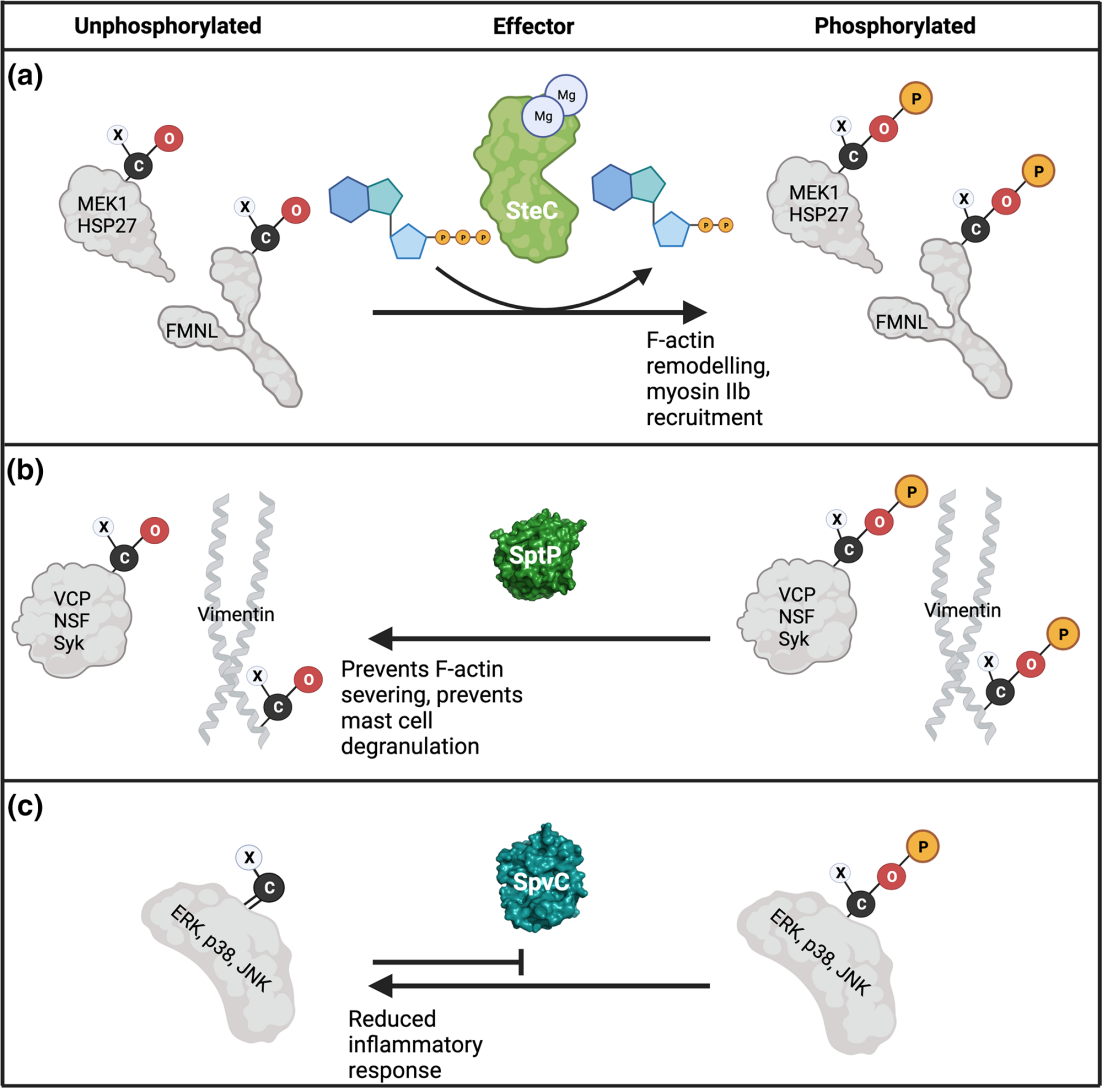
Enzymatic reactions involving protein phosphorylation. (a) SteC is a *Salmonella* effector kinase that has not been structurally characterized. It has three identified host substrates, MEK1, HSP27 and FMNL 1/2. Its kinase activity leads to actin polymerization in the host cytosol. (**b**) SptP is a phosphatase with four identified host substrates, VCP, NSF, Syk and vimentin. Its dephosphorylation of these substrates prevents F-actin severing and mast cell degranulation. (**c**) SpvC has a non-canonical phosphothreonine lyase activity meaning it is able to irreversibly dephosphorylate phosphothreonine residues by severing the Cβ–Oγ bond, requiring a double bond to form between the Cβ and Cα. This is in contrast to the O–P bond which is formed by kinases (**a**) or broken by phosphatases (**b**) and makes the threonine residue unable to reaccept a phosphate moiety. Single letters are used to represent carbon (C), oxygen (O) and phosphate (P). Image created with BioRender.com.

**Fig. 3.**
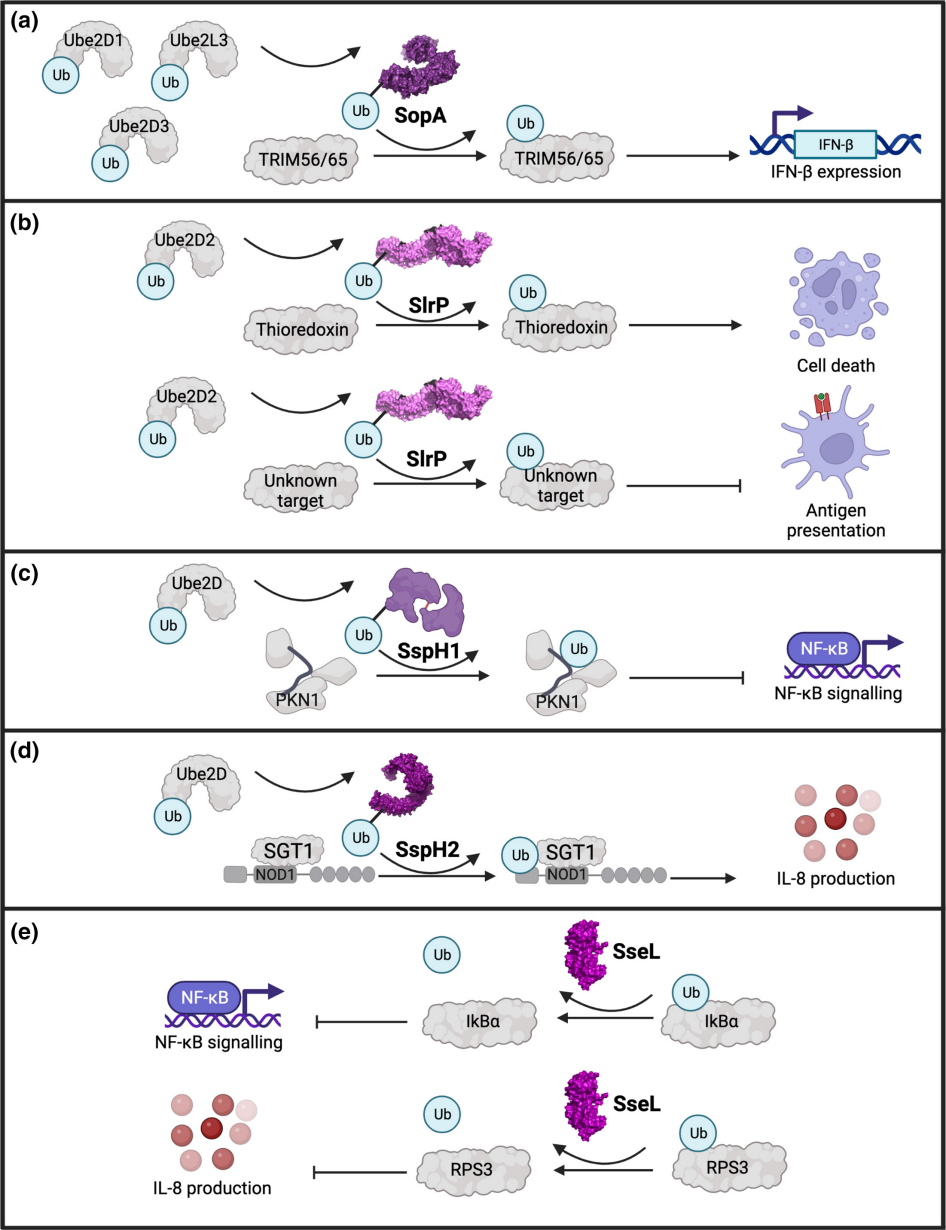
Mechanisms of host manipulation by *Salmonella* effectors through ubiquitylation. (a) SopA mimics the activity of a host HECT E3 ligase and ubiquitylates TRIM56 and TRIM65, leading to increased IFN-β gene expression. The ubiquitin molecule is transferred from a host E2 ligase (Ube2D1/Ube2D3/Ube2 L3) and a temporary bond is formed with SopA. (**b**) SlrP is a novel E3 ligase (NEL) that ubiquitylates thioredoxin to increase cell death and may ubiquitylate an unknown target to inhibit antigen presentation of dendritic cells. The E2 conjugating enzyme involved in the ubiquitin transfer is Ube2D2. (**c**) SspH1 is an NEL that facilitates binding of ubiquitin to PKN1 following transfer from the E2 Ube2D leading to suppression of NF-κB signalling. (**d**) SspH2 is an NEL and following transfer of a ubiquitin molecule from the E2 Ube2D, interacts with SGT1 and NOD1 resulting in the ubiquitylation of NOD1, causing an increase in IL-8 production. (**e**) SseL is a deubiquitylating enzyme (DUB) which removes ubiquitin from IκBα and RPS3 and causes a reduction in NF-κB signalling and IL-8 production respectively. Ubiquitin molecules are depicted as light blue circles containing the text ‘Ub’. Curved arrows demonstrate a transfer of ubiquitin molecules. Bar-headed lines demonstrate inhibition while arrows indicate facilitation/increase in a downstream effect. Human names for the E2 conjugating enzymes have been used throughout. Image created with BioRender.com.

**Fig. 4.**
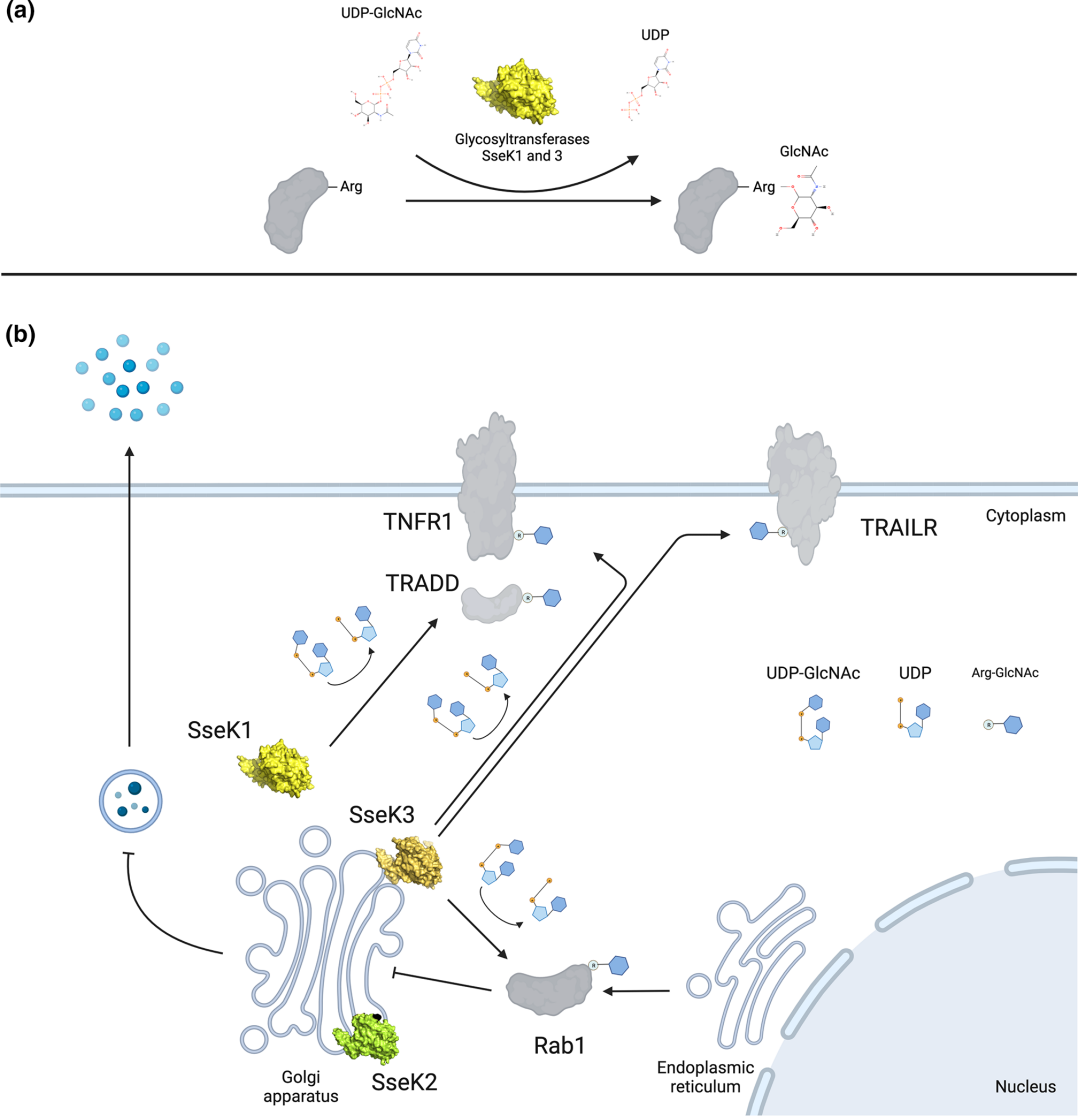
The molecular basis of arginine GlcNAcylation. (a) *Salmonella* effectors SseK1 and SseK3 utilize UDP-GlcNAc to catalyse Arg-GlcNAcylation on host proteins (grey). (**b**) During *Salmonella* infection, SseK1 localizes to the host cell cytosol and modifies the mammalian signalling protein TRADD, while Golgi-localized SseK3 modifies TNFR1 and TRAILR. Thereby, SseK1 and SseK3 target the TNF and TRAIL signalling pathways in host cells. SseK3 also targets Rab1, which mediates host protein transport from the endoplasmic reticulum to the Golgi, thus interfering with host protein secretion. SseK2 localizes to the Golgi, though a host substrate is yet to be identified. Image created with BioRender.com.

**Fig. 5.**
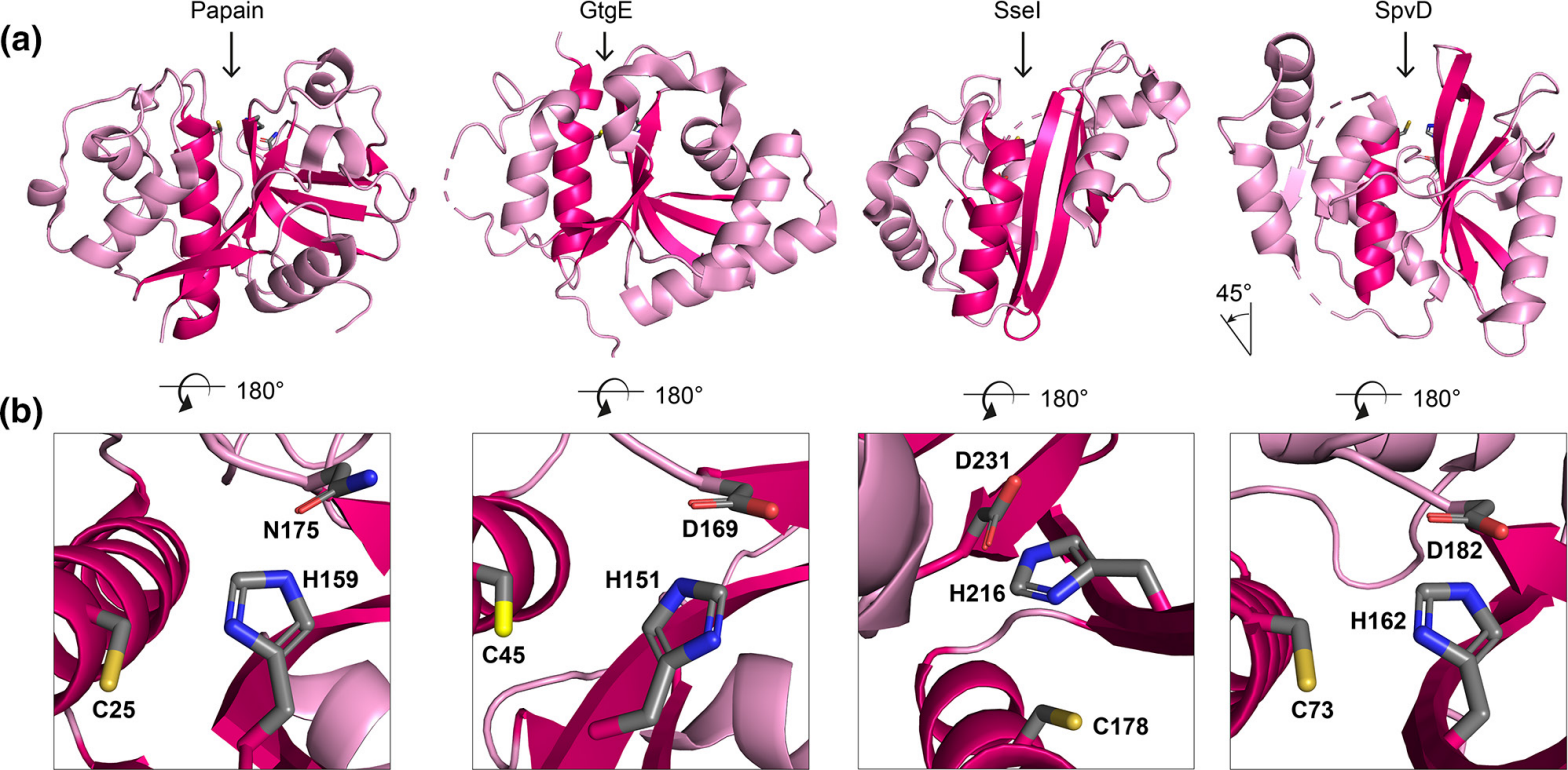
Structural alignment of cysteine proteases GtgE, SseI and SpvD with papain-like folds. (a) Structure of papain (pdb: 1ppn) compared to that of *Salmonella* effectors GtgE (pdb: 5kdg), SseI (pdb: 4g29) and SpvD (pdb: 5lq6). Structures are aligned to the catalytic cysteine of papain (Cys25). The papain-fold motif is highlighted in dark pink and comprises an α-helix containing the catalytic cysteine residue and an anti-parallel β-sheet. The arrow indicates the location of the catalytic cleft/active centre. The structure of SpvD was rotated by −45° along the *xy*-axis for better comparability. (b) Zoom into the active centre of the cysteine proteases shown in (a). Residues of the catalytic triad are shown as sticks and atoms were coloured as follows: carbon – grey, oxygen – red, nitrogen – blue, sulphur – yellow. In contrast to the catalytic triad of GtgE, SseI and SpvD, which is made up of cysteine, histidine and aspartate, that of papain contains an asparagine residue instead of aspartate.

## Biochemical modification of host proteins by *Salmonella* effector proteins

### Phosphorylation

The dynamic process of protein phosphorylation represents one of the most abundant PTMs found in eukaryotes. For canonical protein phosphorylation, kinases mediate the transfer of a phosphate group onto a target serine, threonine or tyrosine residue. Individual kinases can phosphorylate between one and a few hundred phosphorylation sites and with at least 518 annotated human protein kinases it is perhaps unsurprising that up to two thirds of the human proteome can become temporarily phosphorylated [18]. At least 500 host proteins exhibit an altered phosphorylation status upon infection with *Salmonella *, highlighting the importance of phosphorylation in host cell responses to infection [19, 20]. This also represents an opportunity for manipulation by *Salmonella* effectors, either through the direct addition or removal of the phosphate group (discussed in this section, Fig. 2) or through indirect mechanisms including acetylation of kinases (see 'Acylation') and adaptor-mediated reprogramming of a host kinase [see 'Adaptors'].

#### SteC: An effector kinase

SteC is the only recognized *Salmonella* effector kinase and was recently reviewed in depth [21]. With only 28 % amino acid sequence identity to the human serine/threonine kinase Raf1 and lack of several highly conserved residues or motifs found in classical eukaryotic kinases it is remarkable that SteC demonstrates kinase activity [22]. To date, three host substrates, MEK1 [23], HSP27 [20] and FMNL1 [24], have been described (Fig. 2a). MEK1 is activated via serine phosphorylation by upstream activator kinases, including Raf1. Whereas Raf1 phosphorylates MEK1 at S_218_/S_222_ residues, SteC phosphorylates MEK1 at the non-canonical S_200_ residue. This induces MEK1 auto-phosphorylation at S_218_/S_222_ and initiates downstream activation of extracellular signal-regulated kinase (ERK) signalling independent of Raf1 [23]. PAK phosphorylation of MEK1 at S298 also triggers MEK1 autophosphorylation and subsequent activation, suggesting that SteC exploits plasticity in the mechanism by which MEK1 is activated [25]. Whether this form of activation changes the activity, substrate repertoire or negative regulation of MEK1 awaits further investigation. SteC-mediated phosphorylation of MEK1, HSP27 and FMNL1 have been implicated in the reorganization of F-actin and recruitment of myosin IIb [21–23]. The significance of this for *Salmonella* survival and replication remains unclear given wild-type, but not kinase mutant, SteC reduces *Salmonella* replication within epithelial cells and macrophages, as well as in sensitive virulence tests in mice [23].

#### SptP: A dual phosphatase and GTPase activating protein (GAP)

The T3SS-1 effector SptP is a 60 kDa protein with dual enzymatic activity; the N-terminal domain (residues 167–290) is a GTP activating protein (GAP) and the C-terminal domain (residues 280–543) functions as a tyrosine phosphatase [26] (Fig. 2b). The phosphatase domain exhibits both high sequence identity and structural similarity with *Yersinia* spp. effector YopH and human protein tyrosine phosphatases such as PTP1B [26–29] and contains the conserved catalytic cysteine residue, SptP_C481_ [27, 30]. The GAP domain demonstrates high sequence and structural similarity to *Yersinia* spp. effector YopE and *Pseudomonas aeruginosa* ExoS [26, 31, 32].

What then is the function of this dual enzyme? During invasion, SopE, a guanine exchange factor (GEF), activates Rho GTPases to generate GTP-bound Cdc42 and Rac1 that drive actin polymerization to generate the membrane ruffles that mediate bacterial entry into the cell [33, 34]. The GAP domain of SptP inactivates Cdc42 and Rac1 to counteract the activity of SopE and restore the cortical actin cytoskeleton after internalization [35–37]. SptP activity depends on a conserved arginine residue (R_209_) that stabilizes the transition state of the active centre like the arginine finger motif of eukaryotic GAPs, even though SptP displays a distinct protein fold with no amino acid sequence identity to eukaryotic GAPs [30, 35]. Interestingly, host-mediated ubiquitylation mediates the differential half-life of SopE (SopE and SopE2: functional analogs of host GEFs of Cdc42) and SptP, so that SopE is eliminated faster via proteasomal degradation, allowing SptP to act longer than SopE [38] thereby explaining the transient nature of F-actin-mediated membrane ruffling.

Several substrates undergo dephosphorylation through the tyrosine phosphatase activity of SptP, implicated in distinct cellular processes. Vimentin is an intermediate filament protein that is recruited to membrane ruffles and undergoes dephosphorylation by SptP [27]. Villin is an actin-binding protein that, when phosphorylated, nucleates G-actin and severs F-actin. Villin is required for optimal *Salmonella* invasion and becomes rapidly phosphorylated by the host kinase c-Src during infection. This is tempered by SptP phosphatase-mediated inactivation of c-Src [39]. In this way, SptP, together with another actin-binding effector SipA, protects F-actin severing, contributing to the tight regulation of actin remodelling during invasion [39]. SptP tyrosine phosphatase activity is also implicated in increased replication rates of *Salmonella* inside SCVs by dephosphorylating the host AAA+ATPase valosin-containing protein (VCP/p97), which aids SCV membrane fusion [40]. In addition to regulating the actin cytoskeleton, SptP dephosphorylates and inhibits at least two proteins required for mast cell degranulation: vesicle fusion protein N-ethylmalemide-sensitive factor (NSF) and tyrosine kinase Syk, thereby supporting *Salmonella* spread within the host [41]. The absence of SptP is also linked to apoptosis [42], albeit not in *S*. Typhi [43].

Finally, both the GAP and tyrosine phosphatase domains of SptP contribute to the downregulation of *Salmonella *-induced activation of the mitogen activated protein (MAP) kinase, ERK. The GAP domain of SptP prevents GTP-bound Cdc42/Rac1 from activating the host serine/threonine kinases Pak1 and Pak3, which are required for Raf1 _S338_ phosphorylation and subsequent ERK activation [44, 45]. How the phosphatase domain contributes is less clear, but sustained ERK phosphorylation occurs in cells infected with the SptP_C481S_ catalytic mutant compared to cells expressing WT SptP [27].

#### SopB: a phosphoinositide phosphatase

Lipid molecules can similarly be phosphorylated for the purposes of signal transduction. SopB (SigD) is an SPI-1 effector consisting of Cdc42-binding N-terminal (residues 29–142) and C-terminal phosphoinositide phosphatase (residues 357–561) domains. The latter dephosphorylates small phosphoinositide phosphate molecules that are implicated in a range of intracellular signalling pathways. Enzymatic activity was first identified through its CX_5_R sequence motif and phosphoinositide activity was demonstrated *in vitro* [46]. Several functions are attributed to its phosphatase activity through analysis of the SopB_C462S_ mutant. SopB’s early role in invasion involves the stimulation of an endogenous exchange factor, SH3-containing GEF (SGEF), activating the Rho family GTPase RhoG, leading to actin reorganization [47]. Then, at the plasma membrane, SopB indirectly induces the phosphorylation of the serine protein kinase Akt [48, 49]. Once bacteria are inside the cell, SopB indirectly promotes phosphatidylinositol 3-phosphate formation on the SCV by the host PI3-kinase Vps34. Vps34 is activated by Rab5, which is recruited by SopB positioned at the SCV [50]. SopB also regulates SCV subcellular positioning through activation of a Rho – Rho kinase (ROCK) – myosin II pathway [51]. SopB’s enzymatic activity presumably causes these cellular events through triggering phosphoinositide flux. This has not been directly demonstrated, although SGEF has a phosphoinositide-binding pleckstrin homology domain essential for its activity [48]. The temporal progression in SopB’s subcellular localization and activity is mediated by ubiquitylation on several lysine residues in its first 120 aa [48], demonstrating how a host-mediated PTM is co-opted to diversify effector function. The N-terminal domain of SopB binds to Cdc42 through structural mimicry of a host guanosine nucleoside dissociation inhibitor [GDI, see 'Guanine nucleotide exchange factors (GEF) and gtpase activating proteins (GAP)'] [52]. Hence, SopB activates a novel Cdc42-MEK1/2 pathway to remodel vimentin around the SCV to facilitate the intracellular replication of *Salmonella* in a phosphatidylinositol phosphatase-independent manner [53]. Therefore, through enzymatic activity, protein–protein interactions and subcellular localization, SopB has evolved to manipulate multiple cellular pathways.

#### SpvC: a phosphothreonine lyase

SpvC is a phosphothreonine lyase that catalyses the irreversible dephosphorylation of MAP kinases. It achieves this through β-elimination of phosphate, i.e. breaking of the Cβ–Oγ bond (rather than the O–P bond usually broken in a dephosphorylation reaction), making the substrate unable to reaccept a phosphate moiety [42, 54, 55] (Fig. 2c). The irreversible nature of this phosphothreonine lyase activity, which was originally described for OspF from *Shigella flexneri* [56] and has now been expanded to include VirA from *Chromobacterium violaceum* [57] and HopAI from *Pseudomonas syringae* [58], is likely to represent a potent mechanism for the regulation of phosphorylation-dependent signalling, even at low protein concentration. Given this, it is perhaps unsurprising that lyase activity is highly specific: domains within OspF, SpvC and VirA mimic a D-motif that mediates protein–protein interactions towards MAP kinases and, in line with this, phosphothreonine lyase activity occurs on the pT-X-pY motif present in ERK, p38 and c-Jun N-terminal kinase (JNK) [42]. Overall, this results in arrest of downstream immune signalling, which is implicated in a reduced inflammatory response to infection and the promotion of systemic *Salmonella* infection *in vivo* [59].

### Ubiquitylation

Ubiquitylation regulates numerous cellular processes including signal transmission, transcription and the cell cycle [60]. The process, which occurs through three well-defined enzymatic steps involving E1, E2 and E3 enzymes (Box 1), was originally described as the formation of an isopeptide bond between the C-terminal glycine residue of a conserved protein called ubiquitin (Ub) to a lysine residue in target proteins. Its versatility as a protein modifier is expanded through the modification of multiple residues on the target protein or through poly-ubiquitin chains formed via the linkage to either one of seven lysine residues on ubiquitin, or the amine group of the N-terminal methionine [61–64]. Recently, the non-canonical ubiquitylation of serine, threonine and cysteine residues has also been described [65]. Remarkably, *Salmonella* that aberrantly enter the cytosol of epithelial cells are subjected to ubiquitylation of their lipopolysaccharide, transiently targeting the pathogen for antibacterial autophagy early in infection [66] (Box 2). Therefore, a relatively small protein (76 aa) can have a huge and diverse impact on the regulation of cell signalling, with the length of the ubiquitin chain and the type of residue(s) that form the links greatly widening the range of molecular reactions that the substrate can undergo [63, 65, 67]. The central role that ubiquitin plays during infection is exemplified by the dynamic ubiquitylation events defined during infection [68] as well as the identification of five *Salmonella* effectors that directly impact host ubiquitylation despite the absence of a mammalian-like system in bacteria (Fig. 3). Four of these are E3 ubiquitin ligases, which are reliant on the host E1 and E2 components for activity, and the fifth, SseL, is a deubiquitylase (DUB), mediating the removal of ubiquitin from substrates. In this section we describe how modulation of ubiquitin-mediated signalling impacts *Salmonella* pathogenesis.

Box 1. **The mechanism of ubiquitylation**

The combined action of the E1, E2 and E3 classes of enzymes whose functions are activation, conjugation and ligation respectively mediate the attachment of ubiquitin molecules to a substrate [61, 62, 64, 65, 320]. Activation occurs through ATP-dependent linking of ubiquitin to a cysteine residue on the E1 enzyme. Following this, the molecule is transferred to the E2 conjugating enzyme through a thioester transfer. Finally, an E3 ligase enzyme facilitates the covalent linkage of the ubiquitin molecule to the substrate protein [60, 61, 321]. Different categories of E3 enzymes accomplish this via two main and distinct mechanisms, with newer mechanisms recently elucidated that also modify non-proteinaceous substrates [322]. For the RING (really interesting new gene) family of E3 ligase enzymes, the RING domain interacts with both the ubiquitin and the E2 enzyme of the E2–Ub complex to allosterically activate the thioester bond, facilitating transfer of ubiquitin from the E2 directly to the target protein. In contrast, the HECT (homologous to the E6AP carboxy terminus) and RBR family of E3 ligases form an intermediate bond directly with the ubiquitin molecule through transthiolation of the active site cysteine followed by the transfer of ubiquitin to the substrate by the ligases [60, 61, 321]. Finally, ubiquitylation is reversible through the action of DUBs that cleave the ubiquitin molecules or chains from substrates to prevent degradation, turn off signalling pathways and recycle ubiquitin [62, 64, 65, 323].

Box 2. **Antibacterial autophagy**

Autophagic degradation is a process involving the breakdown of long-lived proteins, insoluble aggregates, damaged organelles and various other molecules, including pathogens, through envelopment in a double membrane vesicle known as the autophagosome. Subsequent fusion with a lysosome leads to the degradation of the cargo by the acidic environment and presence of hydrolytic enzymes [62, 63, 65, 320, 324]. For the autophagosomal degradation of intracellular bacteria, termed xenophagy, adaptor proteins such as NDP52 [325] and Optineurin [326] form a link between ubiquitylated targets and the forming autophagosome. Dysregulation of this process results in increased *Salmonella* replication in the cytosol of epithelial cells.

#### SopA: an HECT-type E3 ubiquitin ligase

To date, the SPI-1 effector SopA has been shown to contribute to a number of outcomes during *S*. Typhimurium infection, including (i) the invasion of polarized epithelial cells, (ii) stimulation of polymorphonuclear (PMN) leucocyte transepithelial migration and (iii) induction of electrolyte and water secretion [9, 69, 70]. Early investigations found SopA was poly-ubiquitylated by a host RING-E3 ligase named HsRMA1, which targets SopA for proteasomal degradation (Box 3). An *sopA* deletion or HsRMA1 knock-down resulted in fewer vacuole membrane marker LAMP2-negative bacteria, suggesting that SopA may facilitate escape from the SCV [71]. More recent insights, however, have shown that SopA ubiquitylates substrates itself via HECT-like E3 ligase activity [72] (Fig. 3a). *S*. Typhimurium strains expressing a SopA E3 catalytic mutant (C_753_S) were less able to stimulate PMN transepithelial migration in polarized T84 cells compared to native SopA, suggesting its E3 ligase activity contributes to intestinal inflammation [72]. Regarding host targets, SopA enhances the ubiquitylation of host innate signalling mediators TRIM56 and TRIM65, boosting melanoma differentiation-associated (MDA5) protein function and subsequent upregulation of IFNβ expression [73]. Conversely, another study found that SopA targeted both TRIM56 and TRIM65 for proteasomal degradation [74]. While both studies agree on TRIM56 and TRIM65 as SopA interaction partners, the exact basis for these differences in the outcome of SopA activity requires further investigation. Further evidence suggests the E3 ligase activity of SopA contributes to intestinal inflammation, whereby *S*. Typhimurium strains expressing a SopA E3 catalytic mutant (C_753_S) were less able to stimulate PMN transepithelial migration in polarized T84 cells compared to native SopA [72]. NleL, an *Escherichia coli* SopA homologue, also displays eukaryotic-like HECT E3 ubiquitin ligase activity via the common E2 conjugating enzyme (Ube2L3), but NleL lacks the residues required for SopA–TRIM interaction, suggesting diversification of function [74–77].

Box 3. **Ubiquitin in proteasomal degradation**

Proteasomal degradation is vital to the turnover of proteins in the cell and predominantly degrades short-lived ubiquitylated proteins involved in regulatory cellular processes in an ATP-dependent process [63, 324, 327]. The 26S proteasome, located in the cytosol or nucleus, represents a major pathway for ubiquitin-mediated proteolysis, often targeting proteins with K_48_-linked polyubiquitin chains [60, 328, 329]. The 19S subunit of the 26S proteasome serves a ‘gatekeeper’ function to control entry of proteins. In contrast, the 20S subunit forms the chamber of the complex and is the site of substrate proteolysis. Several *Salmonella* effectors are degraded by the proteasome, including SopE and SptP [38] as well as effector targets such as TRIM56 [48].

#### Novel E3 ligases (NEL) in *Salmonella*

*Salmonella* and several other Gram-negative bacteria express members of a distinct family of bacterial ubiquitin ligases referred to as the NEL family, for Novel E3 Ligase. These proteins harbour a structurally unique C-terminal domain with canonical RING or HECT E3 ligases [78–81]. Despite sequence dissimilarity, NELs act as E3 ligases in an HECT-like way by forming an intermediate bond with ubiquitin before transferring the ubiquitin molecule to the substrate and require ubiquitin charging by E2 enzymes [82].

##### SlrP: a leucine-rich repeat NEL ligase

*Salmonella* leucine-rich repeat protein (SlrP) is an effector which belongs to the leucine-rich repeat (LRR) class of proteins and is translocated by both the SPI-1 and SPI-2 T3SSs [79]. SlrP consists of an N-terminal LRR region, the proposed substrate binding site, and a C-terminal NEL domain housing the catalytic residue and a linker region between the two [11, 83]. In standard conformation, SlrP is inactive owing to the LRR region obstructing the NEL region. However, binding to ubiquitin allows the NEL region to be uncovered and exert E3 ligase activity. SlrP interacts with and ubiquitylates thioredoxin, a ubiquitous protein that functions in concert with other enzymes to protect against oxidative stress, as well as enhancing the activity of transcription factors such as NF-κB and p53 [84–86] (Fig. 3b). Another study found SlrP inhibited the reducing activity of thioredoxin, but this activity was only partially dependent on SlrP E3 ligase activity [80]. SlrP also ubiquitylates a component of the spliceosome (SNRPD2), a protein complex that removes non-coding sequences from pre-mRNA transcripts, but the impact of this is currently unclear [81]. How the currently known substrates mediate the diverse infection outcomes attributed to SlrP, including (i) a role in promoting *S*. Typhimurium virulence *in vivo*, (ii) increased cytotoxicity in epithelial cells, (iii) inhibition of antigen presentation by dendritic cells and (iv) prevention of host anorexia through inhibition of inflammasome activation, remains unknown [80, 87–89].

##### SspH1 and SspH2 and their divergent targets

Like SlrP, SspH1 and SspH2 are LRR proteins with NEL activity. While SspH1 and SspH2 share 69 % amino acid sequence similarity, their localization and interaction partners suggest they induce divergent cellular effects in host cells [11, 90]. SspH1 is translocated by both the SPI-1 and SPI-2 T3SSs and is localized to the nucleus while SspH2, delivered by the SPI-2 T3SS, migrates to the apical surface of epithelial cells [11, 91, 92]. SspH1 interacts with the serine/threonine protein kinase N1 (PKN1) through its N terminal LRR domain, with SspH1-mediated ubiquitylation targeting PKN1 for proteasomal degradation [93, 94] (Fig. 3c). PKN1 is implicated in androgen receptor signalling and is associated with macrophage stimulation and bacterial immunity [95–97]. SspH1 reduces inflammatory gene expression stimulated by NF-κB in epithelial cells through its LRR domain [92, 94, 98]. Although catalytically active PKN1 inhibits NF-κB stimulated gene responses [99], no reliable association between the ubiquitylation and degradation of PKN1 by SspH1 has been linked to the suppression of NF-κB signalling by the effector.

SspH2 targets the NOD-like receptor (NLR) protein Nod1 (Fig. 3d). Presence of the Nod1 chaperone SGT1, which is essential for Nod1 function, augments SspH2 ubiquitylation of Nod1 [100–102]. NLRs play a critical role in detection of pathogen-associated molecular patterns (PAMPs) and initiation of inflammatory responses to promote host defence [103, 104]. Counterintuitively, SspH2 augments the activity of Nod1 in an SGT1-dependent manner, resulting in increased production of IL-8 in infected epithelial cells.

In addition to *Salmonella *, LRR–NEL-containing proteins, referred to as the IpaH family, occur in the gastrointestinal pathogens *Shigella* and enteroinvasive *E. coli* (EIEC) [91, 98, 105, 106]. Some of these have distinct functions, with IpaH9.8 targeting members of the interferon-induced GTPase family of guanylate-binding proteins (GBPs), subverting their function to promote *Shigella* motility and cell-to-cell spread [107]. There is also evident family expansion and substrate diversification of these proteins in invasive *Escherichia* species from non-primate hosts [106].

### SseL: a deubiquitylating enzyme

SseL, a member of the CE clan of cysteine proteases, is the only known *Salmonella* DUB. It exhibits specificity for ubiquitin over other ubiquitin-like modifiers, with a preference for K_63_-linked chains [108, 109]. This suggests a putative role in the regulation of infection-associated signalling that is often mediated via K_63_-linked ubiquitin-regulated protein–protein interactions. Structural analysis of SseL revealed an N-terminal VPS-27, Hrc and STAM (VHS)-like domain. In eukaryotes, the VHS domain is involved in vesicular trafficking, sometimes via ubiquitin binding. Indeed, the VHL domain of SseL constitutes a ubiquitin binding site which is required for the subcellular localization of SseL to the SCV and tubular connections to the SCV called *Salmonella *-induced filaments (SIFs), presumably directing SseL activity at these locations [108].

Functionally, the direct targets of SseL DUB activity are not well defined; early reports suggested SseL targeted NF-κB signalling mediators to prevent downstream inflammatory signalling in both murine and avian macrophage models [110, 111]. However, conflicting reports have emerged on its ability to curb NF-κB signalling through IκBα deubiquitylation [109, 112]. SseL also interacts with, deubiquitylates and inhibits nuclear ribosomal protein S3 (RPS3), a host protein that guides the NF-κB complex to specific gene promoter sites for maximal expression of proteins such as IL-8 [113–115] (Fig. 3e). However, the study only used ectopically expressed SseL [115], and whether RPS3 represents an SseL target under physiological conditions awaits investigation. SseL shares sequence similarity to two cysteine protease DUBs originating from *Chlamydia trachomatis *, *Chla*Dub1 and *Chla*Dub2 [116, 117], as well as ElaD from *E. coli *, which is orthologous to SseL and exhibits DUB activity *in vitro* [118]. *Chla*Dub1 impairs NF-κB activation [116] but the function of *Chla*Dub2 and ElaD, as well as numerous other bacterial DUBs, remain poorly characterized under physiologically relevant settings.

SseL is also required for the deubiquitylation of cytosolic protein aggregates or aggresome-like induced structures (ALIS), formed in a T3SS-dependent manner during infection [119]. Although the content and implications of ALIS remain unclear, SseL counteracts this ubiquitin-dependent selective autophagic response mounted by the host, via its deubiquitylation activity [119]. Finally, SseL’s interaction with host protein OSBP, which results in no apparent ubiquitin-mediated regulation of OSBP, is discussed in 'SseL and its interaction with OSBP'.

### Glycosylation

Protein glycosylation is the covalent attachment of glycan moieties, including carbohydrates or sugars, to a protein. *N*-glycosylation refers to the attachment of glycans to the side-chain nitrogen atoms of asparagine whereas *O*-glycosylation is their attachment to the side-chain oxygen atoms of hydroxyl amino acids such as serine or threonine. In mammalian cells, *N*-acetylglucosamine (GlcNAc) is transferred from an activated uridine-diphosphate donor substrate (UDP-GlcNAc) to the hydroxyl group of serine and threonine by a glycosyltransferase, termed GlcNAcylation. Remarkably, a family of bacterial effectors catalyse the addition of GlcNAc to arginine residues with an *N*-glycosidic linkage (Fig. 4). First described for enteropathogenic *E. coli* (EPEC) effector NleB1, this pathogen-specific modification is irreversible by the host cell and in the case of NleB1 inhibits proinflammatory immune responses and host cell death [120–122].

#### SseK1, SseK2 and SseK3: related arginine-glycosyltransferases

Three *Salmonella* glycosyltransferases, SseK1, SseK2 and SseK3, which are translocated via the SPI-2 T3SS, share high sequence similarity with NleB1 [123, 124]. According to their structures, NleB and SseK effectors are classified as type A family (GT-A) glycosyltransferases. Each modifies a critical arginine residue of target proteins with a single GlcNAc moiety. Similar to other structurally characterized bacterial glycosyltransferases, this group of effectors all contain a conserved metal-coordinating DXD motif (SseK1 _223_DAD_225_; SseK2 _239_DAD_241_; SseK3 _226_DAD_228_) that is indispensable for mediating their enzymatic activity [125–127]. Several studies have investigated the sugar transfer mechanism of these glycosyltransferases. A retaining sugar transfer mechanism was proposed for SseK3 [126] and SseK1 [125], resulting in an alpha-anomeric glycosidic linkage. Retaining glycosyltransferases are thought to operate through stabilization of an oxocarbenium-like transition state [128] with different mechanisms proposed [128–131]. For SseK3, the glutamate side chain of SseK3_E258_ was proposed to act as the intramolecular nucleophile in the first step of a double-displacement mechanism [126]. However, the corresponding residues, SseK1_E255_ and SseK2_E271_, are not essential for catalysis [125]. Therefore, for SseK1 at least, a so-called S_N_i, single displacement reaction, is proposed, where the β-phosphate of the UDP formed in the reaction acts as the catalytic base to activate the acceptor arginine [125]. Interestingly, NleB, similar to mammalian *O*-GlcNAc transferase (OGT) [132], acts via an inverting glycosyltransferase mechanism, so that NleB modified substrates displaying a beta-anomeric configuration [127].

Upon translocation into host cells, the SseK effectors exhibit distinct subcellular localization patterns and target different host substrates (Fig. 4). Both SseK2 and SseK3 localize to the Golgi, whereas SseK1 localizes to the cytosol of host cells [133]. The development of an mAb specific for the Arg-GlcNAc modification [134] has aided identification of host substrate specificity of the SseKs, but the findings have been conflicting [120, 133, 135, 136]. What is clear is that overexpression of the NleB1/SseK effectors results in inauthentic or promiscuous modification of many host and bacterial proteins [122], whereas native expression results in specific modified targets. For example, during *Salmonella* infection, natively expressed SseK1 modifies the death receptor signalling protein TRADD, while SseK3 modifies receptors of the mammalian TNF superfamily, including TNFR1 and TRAILR [136]. Both SseK1 and SseK3 function cooperatively to inhibit NF-κB signalling and necroptotic cell death when translocated into macrophages, in an Arg-GlcNAc-dependent manner [133, 137]. While some enzymatic effectors have limited host targets this is not the case for SseK3. Two recent studies found that during *Salmonella* infection *in vitro*, SseK3 modified several small Rab (GTPases) on arginine residues, including Rab1, Rab5 and Rab11 [138, 139]. Rab1, which mediates vesicle transport from the endoplasmic reticulum (ER) to the Golgi, was targeted by SseK3 in its switch II region, reducing protein secretion [138]. Interestingly, Rab1A is inhibited by SseF and SseG, resulting in inhibition of Rab1A-mediated autophagy, supporting the formation of a replicative niche [140]. Furthermore, Rab5 is recruited to the early SCV by SopB [50] and activated by SopE (a GEF) [141]. However, the implications of this effector cross-talk on host Rab proteins remains unclear.

Interestingly, the substrate specificity of the NleB/SseK family of effectors is finely tuned by a single residue close to the catalytic core (Y_284_ in NleB1) [142]. Whereas Y_284_ dictates a relatively broad substrate specificity by NleB, SseK1 has a narrow substrate range. Amino acid substitution of S_286_ in SseK1 or N_302_ in SseK2 with Y promoted Arg-GlcNAc modification of FADD and DR3 proteins, respectively [142]. Interestingly, definitive substrate identification for native SseK2 has so far been elusive, suggesting SseK2, much like NleB2 from EPEC [143], may use an alternative sugar for arginine modification of host proteins. Nevertheless, expression of either SseK1_S286Y_ or SseK2_N302Y_, in a triple *sseK* null background, but not WT SseK1 or SseK2, resulted in complete restoration of the replication defect observed for the triple *sseK* mutant in RAW macrophages. This suggests that with artificial expansion of the substrate repertoire, *Salmonella* no longer requires all three SseK effectors for its optimal intracellular replication.

Besides targeting substrates of host cells, SseK1 and SseK3 undergo self-GlcNAcylation which is critical for their enzymatic activity [144]. Furthermore, SseK effectors are reported to modify an array of bacterial proteins, but whether this occurs in intact bacteria or represents an experimental artefact awaits further experimentation [125, 136, 144, 145].

Several *in vivo* studies have explored the role of the SseKs in murine infection, albeit with mixed results [123, 124, 139, 146–149]. Clearly more studies are required to clarify the contribution of SseK effectors to *Salmonella* pathogenicity *in vivo*, as well as continued efforts to further define the physiological substrates of each effector.

### Ribosylation

ADP-ribosylation is the enzymatic addition of ADP-ribose to a protein substrate catalysed by poly (ADP-ribose) polymerases (PARPs) with the use of NAD^+^ (Fig. 1). It plays a critical role in numerous host cellular processes including DNA repair, modulation of cell signalling pathways and initiation of a form of programmed cell death known as parthanatos [150]. *Salmonella* encodes two ADP-ribosyltransferase effectors, SpvB and SopF, each of which subverts host innate responses.

#### SpvB: an actin targeting ADP-ribosyltransferase

SpvB is an effector encoded within the *spv* operon on *Salmonella* virulence plasmids, which plays a critical role in mediating systemic *Salmonella* infection in mice [151, 152]. SpvB, identified as an ADP-ribosyltransferase via PSI-blast analysis [153], harbours a conserved NAD binding site in the C-terminal domain to perform ADP-ribosylation [152]. Structural analysis demonstrates a canonical fold as seen in other bacterial ADP-ribosyltransferases [154]. During *Salmonella* infection, SpvB is translocated via the SPI-2 T3SS and targets actin within infected host cells [11, 152, 155]. SpvB modifies R_177_ of actin to interfere with ATP hydrolysis, thereby inhibiting actin polymerization [156, 157]. The depolymerization of actin via SpvB eventually results in the loss of filamentous actin content and downregulates SIF biogenesis in infected cells [155, 158]. Interestingly, the effect of SpvB on host cell actin antagonizes the activity of another *Salmonella* effector SteC, which promotes the formation of a meshwork of F-actin around SCVs at an earlier stage of infection [22]. Therefore, the activity of SpvB is tightly regulated in a time-dependent manner.

SpvB has functions beyond inducing morphological changes of actin, with the expression of SpvB activating caspase-3 and inducing apoptosis in macrophages [159, 160]. SpvB also induces the disassembly of P-bodies, which are cytoplasmic domains involved in post-transcriptional regulation processes [161] and contributes to the disruption of the intestinal cell barrier, contributing to *Salmonella* dissemination in infected hosts [162]. Additionally, SpvB mediates the downregulation of NRF2 and modulation of intracellular iron homeostasis [163] and NF-κB signalling via antagonizing IKKβ [164]. However, whether these functions require the enzymatic activity of SpvB awaits further study. Importantly, several studies have indicated a critical role for SpvB in colonization and virulence of *Salmonella in vivo* [148, 165–167]. In summary, SpvB has one clearly defined host substrate, yet hijacks multiple host cell processes to promote *Salmonella* virulence.

#### SopF: ADP-ribosylation to inhibit autophagy

Recently, a *Salmonella* SPI-1 T3SS translocated effector SopF was identified as a novel ADP-ribosyltransferase [168, 169]. Although the sequence of SopF shows no homology to previously identified ADP-ribosyltransferases, SopF bears an ‘I-Y-E’ catalytic triad, which is similar to the catalytic triad found in diphtheria toxin-like ADP-ribosyltransferase [170]. ADP-ribosylation factor (ARF) GTPases bind SopF, and the N-terminal domain of ARF1 was required to activate SopF [170]. During *Salmonella* infection, SopF is translocated into host cells to modify Q_124_ of ATP6V0C in the V-ATPase. Such modification prevents recruitment of ATG16L1 by V-ATPase to the damaged SCVs thereby inhibiting LC3 lipidation, which is critical in initiating antibacterial autophagy [169]. In another study, SopF was shown to bind to phosphoinositides and was required to promote SCV membrane stability [171]. By targeting the V-ATPase–ATG16L1 axis of host cells, SopF functions as a broad-spectrum antibacterial autophagy inhibitor without affecting canonical autophagy [169]. Notably, a *ΔsopF S*. Typhimurium mutant exhibited attenuated virulence in mice compared to the wild-type strain, supporting its role in antagonizing the host immune response to infection [169].

### Acylation

Acylation is the addition of an acyl group (R-C=O) to a protein or lipid substrate. When cholesterol is esterified, the carboxylate group of a fatty acid (acyl chain) is activated with thio-coenzyme A to form an acyl-CoA. When this reacts with the hydroxyl group of cholesterol, a cholesterol-ester is formed.

#### SseJ – an acyltransferase

SseJ is an SPI-2 translocated acyltransferase effector [50]. Amino acid sequence analysis of SseJ identified both a ‘GDSL’ motif and catalytic triad composed of serine (S_151_), aspartic (D_247_) and histidine (H_384_) residues, common to GDSL-type lipases [172, 173]. The effector exhibits both phospholipase A and glycerophospholipid:cholesterol acyltransferase (GCAT) activity, as well as deacylase activity [172, 174, 175]. Upon translocation, SseJ associates with the host GTP-bound small GTPase RhoA in order to become enzymatically active and perform membrane cholesterol esterification, which is the transfer of acyl chains from glycerophospholipids onto free cholesterol [175–177]. Subcellular localization studies revealed that SseJ localized to SCVs and SIFs [178, 179]. Consistent with its localization pattern, SseJ regulates SCV membrane dynamics and the biogenesis of SIFs [179–181]. The importance of SseJ in membrane dynamics is highlighted by the deletion of SseJ in a *sifA* mutant background rescuing the unstable nature of *sifA* mutant SCVs [179, 182]. Also, ectopic expression of SifA and SseJ in HeLa cells induces formation of tubular structures, which mimic tubules induced upon *Salmonella* infection [183]. Thirdly, together with SseL, SseJ recruits oxysterol-binding protein-1 (OSBP) to the SCV and without SseJ and SseL, SCVs are unstable [184]. Fourth, a recent study revealed that the catalytic activity of SseJ is required to suppress the expression of host cholesterol transport protein ABCA1 and induce cholesterol accumulation in infected macrophages [185]. Therefore, SseJ, through its acyltransferase activity and in concert with SseL (see 'SseL and its interaction with OSBP') regulates the biogenesis and integrity of SCVs and SIFs.

### Acetylation

Acetylation is a PTM conserved across all domains of life and is carried out by *N*-acetyltransferases. The *Yersinia* outer membrane protein J (YopJ) was the founding member of a superfamily of effectors found in diverse plant and animal pathogens that display unique acetyltransferase activity to potentiate infection [186], referred to as the YopJ family. Despite sharing a conserved catalytic triad that is identical to that of the C55 family of cysteine proteases, they modify host target proteins by acetylating specific serine, threonine and/or lysine residues. Several *S. enterica* serovars encode AvrA, an acetyltransferase effector of the same superfamily that is translocated by both the SPI-1 and SPI-2 T3SS machinery.

#### AvrA: an anti-inflammatory acetyltransferase

All members of the YopJ family of acetyltransferases, including AvrA, require two co-factors for their activity: acetyl-CoA (AcCoA) as the source of the acetyl group and inositol hexakisphosphate (IP6) [187]. Mutation of the core catalytic cysteine, AvrA_C172A_, abolishes its autoacetylation, while individual mutation of residues E_142_, I_179_ and C_186_ mediate a reduction in the acetyltransferase activity of AvrA [188]. Of note, despite early reports that AvrA might act as a deubiquitylase, it is now considered a dedicated acetyltransferase [108].

What then are the targets of AvrA’s acetyltransferase activity? Initially described to inhibit pro-inflammatory NF-κB signalling [189], subsequent studies in both *in vitro* and *in vivo Salmonella* infection models revealed that AvrA specifically inhibits c-Jun N-terminal kinase (JNK) signalling via acetylation of a key threonine residue within the MAPK kinases (MAPKKs), MKK4 and MKK7 [190, 191]. Analysis of the allelic L_140_ variant of AvrA demonstrated that this residue is essential for JNK suppression, as the variant functions to prohibit interaction with MKK4/7 [192]. Through this mechanism, AvrA simultaneously reduces JNK-mediated inflammatory and apoptotic responses in both intestinal epithelial cells and macrophages, probably to promote a replicative niche. Of note, unlike YopJ itself, AvrA does not inhibit the ERK MAPK pathway and, instead, ERK2 interacts with and phosphorylates AvrA, inhibiting its activity [190]. Finally, several other functions for AvrA have been reported, with poorly defined mechanisms [188, 193–200]. Most of these studies use strains with a PhoP constitutive mutation (phoP^c^). With the expression of more than 40 proteins altered [201], this renders the bacteria attenuated for virulence and survival in macrophages, and therefore the physiological significance of the findings are unclear.

### Proteolysis

Proteolysis is the enzymatic breakdown of proteins following hydrolysis of peptide bonds by a protease. It results in an irreversible alteration of the protein and is essential for maintaining cellular homeostasis as well as regulating cell signalling. The irreversible nature of proteolysis represents a potent method by which pathogens can alter host-mediated signalling [202]. Several *Salmonella* effectors are designated as proteases, encompassing two classes: the cysteine proteases (GtgE, SpvD and SseI) and the zinc-metalloproteases (GtgA, GogA and PipA) [11]. Cysteine proteases use a cysteine residue for nucleophilic attack of the target peptide bond. Meanwhile, zinc metalloproteases use an activated water molecule as the nucleophile. Other proteases include serine, threonine, aspartic and glutamic proteases, and asparagine peptide lyases. However, *Salmonella* is not known to encode any of these peptidases as effectors. The *Salmonella* DUB, SseL, a cysteine protease which specifically cleaves a ubiquitin moiety is addressed in the ubiquitin-specific section above ('Ubiquitylation').

#### Cysteine proteases

GtgE, SpvD and SseI represent cysteine proteases, with similarity in their catalytic triad and papain-like fold (Fig. 5). The structural similarity and ordering of catalytic-site residues places each of these in the CA clan. Despite this, the proteins are not redundant and indeed different functions and substrates are described, with SseI acting as a deamidase, in which the amide group of glutamine is converted to glutamic acid. Therefore, as for eukaryotic proteins, similar catalytic triads can result in different biochemical activity and ultimately function.

##### GtgE: a GTPase-targeting protease

GtgE, which is required for replication and virulence *in vivo*, consists of a papain-like fold and catalytic triad comprising C_45_, H_151_ and D_169_ [203] with the triad side chains requiring minor adjustments to resemble that of an active enzyme state [204] (Fig. 5). GtgE targets three members of the Rab family of GTPases: Rab29, Rab32 and Rab38, cleaving after G_59_ in Rab32 which results in an inactive GTPase conformation [205]. Rab proteins are found in GTP-bound active states and GDP-bound inactive states with the switch I and switch II regions of Rab proteins moving from a disordered state when inactive to a well-defined structure in the active form. This change, and in particular the positioning of F_88_ of Rab32, allows for the remarkable discrimination of Rab32:GDP from Rab32:GTP so that GtgE displays preferential cleavage towards inactive GDP-bound Rab32 [206]. This finding also explains why Rab32:GTP inactivation by the GAP SopD2 ('SopD and SopD2: Two effectors with GAP activity') is required for the activity of GtgE [206, 207], highlighting another example where two *Salmonella* effectors functionally cooperate. Interestingly, GtgE is absent from *S*. Typhi and this contributes to the narrow host range observed in which only humans develop typhoid fever. Remarkably, an *S*. Typhi strain engineered to express *gtgE* is able to replicate within murine macrophages and colonize mice [208], elegantly addressing the importance of serovar-specific effector repertoires.

##### SpvD and SseI: same protein fold, different biochemistries

Despite evident structural similarity between SpvD, SseI (also called SrfH) (Fig. 5) and cysteine hydrolase effectors from other Gram-negative pathogens, each is functionally distinct. OspI from *Shigella flexneri* deamidates Ube2N (Ubc13), a ubiquitin-conjugating enzyme, to inhibit TRAF6 [209] whereas AvrPphB, from the plant pathogen *Pseudomonas syringae *, targets plant kinases for cleavage [210]. This probably reflects differences in substrate binding surfaces and/or orientation of the catalytic triad and means that identifying a putative biochemical activity through structural homology is not always sufficient to elucidate effector function. Indeed, identifying protease targets requires significant effort and while functionally, SpvD inhibits NF-κB in a catalytically dependent manner [211] it is unclear if this is via its only known interaction partner exportin-2 [212]. Furthermore, despite evidence that recombinant SpvD cleaves a C-terminal peptide of ubiquitin (RLRGG) fused to aminoluciferin, SpvD does not cleave full-length di-ubiquitin substrates [211]. Intriguingly, a single-nucleotide polymorphism at position 161, identified between serovars of *S.* Typhimurium (R_161_) and *S*. Enteritidis (G_161_), altered the catalytic activity of SpvD. Given its proximity to the catalytic site, this probably alters substrate access to the active site, with arginine occupying a larger space. Whether this changes the substrate specificity requires further investigation, but SpvD_G161_ elicited greater *Salmonella* virulence in mice compared to strains expressing SpvD_R161_ [211].

SseI is one of several SPI-2 effectors that inhibits dendritic cell migration and promotes long-term systemic infection [213, 214]. Structural resolution of the catalytic domain of SseI revealed that it resembles a cysteine protease or deamidase (Fig. 5) [215]. More recently, SseI has been demonstrated to convert the glutamine residue Q_205_ of GNa_i2_ from the heterotrimeric G protein family to glutamic acid, confirming deamidase activity. This results in constitutive activation of GTP hydrolysis and this loss of polarized activation/deactivation might explain how SseI blocks directed dendritic cell migration towards chemokines [216]. Furthermore, SseI shows similarity to a handful of other SPI-2 effectors in its N-terminal domain and, like SspH2, SseI is localized to the plasma membrane via S-palmitoylation of C_9_, which is important for its function [217]. This therefore exemplifies how individual domains of an effector, as well as specific host-mediated PTM, can be essential for accurate effector localization and function. To this end, it is at the plasma membrane that SseI interacts with IQGAP1 (IQ motif containing GTPase activating protein 1), a host protein that functions as a modulator of cell migration [213]. Finally, as seen for SpvD and GtgE, serovar-specific phenotypes have been described for SseI. In ST313 *S*. Typhimurium isolate D23580 [213], which causes systemic bacteraemia in sub-Saharan Africa, *sseI* is a pseudogene. Despite SseI preventing dendritic cell migration, loss of its activity is associated with hyper-dissemination of this serovar of bacteria to the mesenteric lymph nodes [218]. Additionally, an SNP at position 103 controls whether SseI interacts with TRIP6 in a yeast two-hybrid assay [219]. Overall, despite structural similarity in the catalytic triad (Fig. 5), SpvD, SseI and GtgE are unique effectors that are functional and biochemically distinct.

### GogA, PipA and GtgA: Zinc metalloproteases that cleave NF-κB

GogA, PipA and GtgA are a family of *Salmonella* effector zinc metalloproteases that suppress proinflammatory immune responses by cleaving p65 NF-κB [220, 221]. These proteins demonstrate a characteristic HExxH motif, whereby the two histidine residues bind zinc at the active site while the glutamate coordinates a zinc-bound water molecule for nucleophilic attack of the carbonyl group of the substrate [222]. Structural analysis revealed that the binding of GtgA to the N-terminal domain of p65 mimics the NF-κB–DNA interaction with mutational analysis uncovering the basis for specificity of GtgA towards p65, RelB and cRel, but not NF-κB1 and NF-κB2 [223]. This is in contrast to NleC, a homologue from EPEC, which cleaves all five NF-κB family members at a different site [220, 224–231]. It is interesting to note that several Gram-negative bacteria have evolved GtgA/NleC-like peptidases and further work analysing the divergence of substrate selectivity may reveal how effectors from this peptidase family have evolved [232].

### Guanine nucleotide exchange factors (GEFs) and GTPase activating proteins (GAPs)

GTPase proteins are a large family of small well-conserved eukaryotic enzymes that act as molecular switches, governed by their binding with GDP (inactive) or GTP (active) in the switch 1 and 2 regions. Usually only the active form binds and activates downstream effectors. Cycling between inactive and active forms requires catalysis by GEFs (activating) and GAPs (inactivating). GEFs function by eliminating GDP allowing for free GTP to bind, while GAPs function by accelerating GTP hydrolysis to GDP. Guanine nucleotide-dissociation inhibitors (GDIs) sequester the inactive pool of Rho- and Rab-family GTPases, providing another level of regulation in these cases [233, 234]. The importance of this host regulatory system during infection is demonstrated by the numerous examples of GTPases as host targets of effectors in this review alone. For example, SseK3 glycosylates and GtgE cleaves certain Rab GTPases to inactivate them ('Glycosylation' and 'GtgE: a GTPase-targeting protease', respectively). Meanwhile, SopF ('SopF: ADP-ribosylation to inhibit autophagy') and SseJ (SseJ – an acyltransferase') bind GTPases and SseI ('SpvD and SseI: same protein fold, different biochemistries') binds a host GAP without evidence of direct biochemical modification. Furthermore, SifA contains a WxxxE motif classic of GEFs and demonstrates structural mimicry of GTPases to bind its host target PLEKHM2 ('SifA and its role in membrane stability'). Similarly, SopB structurally mimics a host GDI in binding Cdc42 ('SopB: a phosphoinositide phosphatase'). However, in this section we focus on four *Salmonella* effectors closely related either by sequence or function to eukaryotic GEFs and GAPs. The GAP activity of SptP is described above ['SptP: A dual phosphatase and GTPase activating protein (GAP)'].

#### SopD and SopD2: two effectors with GAP activity

The *Salmonella* effector protein SopD is required for complete virulence as a *ΔsopD* mutant exhibits reduced replication *in vitro* and in murine models of infection compared to wild-type *Salmonella* [235]. Early studies indicated that SopD induced inflammatory responses and gastroenteritis in infected hosts cooperatively with other *Salmonella* effector proteins [70, 191]. SopD also contributes to membrane fission and micropinocytosis during *Salmonella* infection together with SopB [236]. More recent discoveries showed that SopD targets small Rab GTPases of host cells, including Rab8 and Rab10, to manipulate host immune responses during *Salmonella* infection [237–239]. Here, SopD demonstrated GAP activity towards Rab10, resulting in recruitment of Dynamin-2 to promote scission of the plasma membrane of infected host cells [237]. In another study, however, SopD induced higher GTPase hydrolysis of Rab8 compared to Rab10 [239]. As active Rab8 recruits phosphoinositide 3-kinase (PI3K) to mediate Akt-dependent anti-inflammatory programmes [240–242], the GAP activity of SopD promoted inflammatory signalling in infected host cells [238]. In addition, SopD demonstrated GEF activity towards Rab8, which contributes to the displacement of Rab8 from its cognate inhibitor (GDI) resulting in Rab8 activation. Importantly, the GEF activity towards Rab8 was independent of its GAP activity [238]. It remains unclear how SopD coordinates antagonistic activities towards Rab8 during infection, and further studies are required to reveal mechanistic details.

SopD2 is thought to have arisen through gene duplication of SopD, given sequence similarity of 43 % and significant structural similarity of the two effectors [243]. SopD2 is an SPI-2 effector which is associated with the localization and dynamics of the SCV and the obstruction of endosomal movement [243–245]. The N-terminal domain of SopD2, which is not homologous with SopD, is essential for its localization at SCVs and endosomes [246]. Targets of SopD2 include Rab7, Rab8, Rab10, Rab32 and Rab34 [207, 243, 247] although the downstream effect of interactions with Rab8 and Rab10 have not been characterized. Rab7 activity is inhibited by direct SopD2 binding and GAP activity and leads to a halt of endosomal trafficking and dissociation with Rab7 effector proteins, potentially preventing the fusion of the SCV with lysosomes and promoting the cell-to-cell spread of bacteria [243]. A proteomic screen revealed that annexin A2 (AnxA2) interacts with SopD2 during infection [244]. Given the ability of AnxA2 to interact with Rab7 and modulate cytoskeletal rearrangement in the host, the authors speculated that AnxA2 assisted in positioning the SCV inside the cell and altered the endocytic pathways to promote intracellular growth; however, this needs validation.

Rab32, in concert with the BLOC-3 complex, contributes to host defence against *Salmonella *, possibly by delivering antimicrobial peptide-containing lysosomal-related organelles to the SCV [207, 248]. In concert with the cysteine protease SPI-2 T3SS effector, GtgE, SopD2 utilizes its GAP activity to target Rab32 to nullify this pathway and restore intracellular infection [248]. Rab34, on the other hand, is involved in the maturation of the SCV and fusion of the SCV with lysosomal components [247]. This study suggests SopD2 exploits the function of Rab34 to enhance the intracellular replication of *S*. Typhimurium, but it remains unclear whether SopD2 GAP activity is required. The effects mediated by SopD2 *in vitro* involve inhibition of endocytic migration as a mechanism to augment intracellular replication of *Salmonella *. This provides an explanation for observations of SopD2 being important for the virulence and replication of *S*. Typhimurium in murine *in vivo* models and intracellular growth in macrophages [235, 249–251]. Further, SopD2 can contribute to suppression of antigen presentation by dendritic cells, either individually or in concert with SPI-2 effectors SspH2 and SlrP [87].

#### SopE and SopE2: functional analogues of host GEFs of Cdc42

The two effector proteins SopE and SopE2 display 69 % amino acid sequence identity and function as GEFs for the rho-GTPase Cdc42 [37, 252]. Both effectors are injected as part of the SPI-1 effector group, localizing to the early SCV to potentiate early intracellular replication [253]. Interestingly, they demonstrate no sequence [254] or structural homology to eukaryotic GEFs [255]. In complex with Cdc42_1-178_, SopE employs novel amino acid residues to interact with the substrate compared to the eukaryotic GEF Tiam1, though the impact of the interaction on Cdc42 switch regions is similar [255].

SopE and SopE2 have divergent substrate specificity, with only SopE demonstrating GEF activity towards Rac1 [254], pointing to a mechanism by which *Salmonella* may tune its effect on host Rho GTPases. SopE also demonstrates GEF activity towards Rab5, binds Rab5 in its GTP-bound state and recruits it to the phagosome where Rab5 is active and promotes fusion with early endosomes. Though Rab5 usually requires prenylation for activity, a Rab5 prenylation-resistant mutant recruited by SopE recovers activity [141]. The short half-life of SopE compared to SptP ['SptP: A dual phosphatase and GTPase activating protein (GAP)'] means SptP reverses SopE Rho GTPase activation after cell entry [36, 38].

### Adaptors

**Fig. 6.**
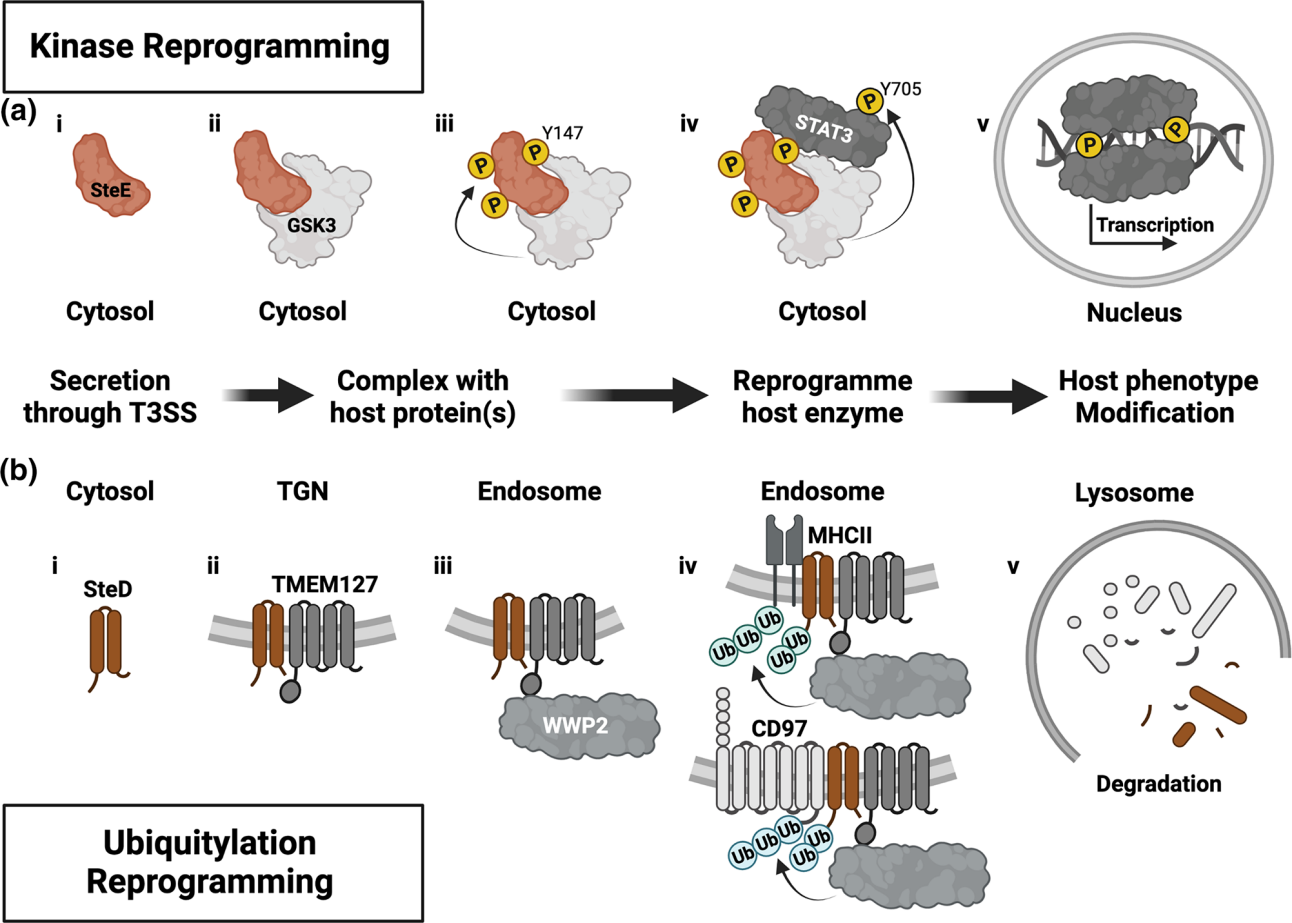
Adaptor proteins that reprogramme host enzymes to new substrates SteE (a) and SteD (b) (both in brown) are (i) translocated to the cytosol through a type III secretion system. (ii) After translocation they interact with host proteins; SteE forms a complex with GSK3 in the cytosol and SteD binds to TMEM127 in the trans-Golgi network (TGN), from where it is transported to an endosome called the MHCII compartment. (iii) GSK3 phosphorylates SteE and E3 ligase WWP2 is recruited to the TMEM127–SteD complex. (iv) phosphorylated SteE recruits STAT3 to the complex and GSK3 phosphorylates the non-canonical substrate STAT3 on tyrosine 705. SteD recruits MHCII or CD97 to the complex and WWP2 ubiquitylates the targets. (v) Phosphorylated STAT3 activates anti-inflammatory pathways and ubiquitylated MHCII and CD97 are targeted for degradation, reducing their cell surface levels and dampening T-cell activation. Image created with BioRender.com.

Numerous *Salmonella* effectors display enzymatic activity and directly target host substrates to modify their function. However, other *Salmonella* effectors lack domains with predicted enzymatic activity; how then do these effectors operate? To compensate for their lack of enzymatic activity, many effectors appear to function as adaptors that facilitate non-canonical interactions between host proteins, one of which is often an enzyme, giving the potential for indirectly modifying multiple host substrates. In this section, we review how various adaptors function to manipulate the host cell during infection. A common theme is the reprogramming of host enzymes towards infection-specific substrates (Fig. 6).

#### SteE: a kinase reprogramming adaptor

SteE is a small protein of 157 aa, translocated through the SPI-I and SPI-2 T3SSs. Mechanistically, SteE drives a change in amino acid specificity in the host serine/threonine kinase glycogen synthase kinase 3 (GSK3), resulting in the phosphorylation of the transcription factor signal transducer and activator of transcription 3 (STAT3) at Y_705_ [256] (Fig. 6). Interestingly, SteE contains a consensus GSK3-binding motif SLPV(p)SP, but instead of phosphorylating this motif, GSK3 is responsible for the phosphorylation of SteE at Y_17_, T_91_, S_141_ and Y_143_, with GSK3-mediated SteE phosphorylation required for STAT3 phosphorylation [256]. As GSK3 is an S/T kinase, one might speculate that phosphorylation of tyrosine residues in SteE and STAT3 requires a conformational change in the GSK3–SteE complex, but this has not been experimentally investigated. Interrogation of the SteE sequence revealed that SteE mimics the cytoplasmic domain of the glycoprotein 130 (gp130), which is involved in JAK-dependent STAT3 phosphorylation [257]. Indeed, this region contains a classical SH2-binding motif, (p)Y_143_xxQ, and its phosphorylation at Y_143_ by GSK3 promotes recruitment of STAT3 as a substrate [256, 257]. STAT3 phosphorylation mediates its activation as a transcription factor, and this results in increased surface levels of IL-4 receptor α and secretion of IL-10 [256, 258]. In this way, SteE drives an anti-inflammatory response in infected cells, counteracting the pro-inflammatory role of TNF⍺ in the granuloma [259] and resulting in the polarization of macrophages to an M2-like anti-inflammatory state that provides a niche for immune evasion, survival and persistence of *Salmonella* Typhimurium [260]. A similar role for SteE in the long-term survival of bacteria was recently observed in *S*. Pullorum-infected chickens [261].

#### SteD: an adaptor that redirects the host E3 ligase WWP2

SteD drives the ubiquitylation of mature major histocompatibility complex class II (mMHCII) by the host E3 ligase WWP2. First, SteD hijacks the host AP1-mediated trafficking pathway through the ER, Golgi and trans-Golgi network to reach MHCII compartments [262], where it binds to mMHCII [263]. SteD interacts indirectly with WWP2 via transmembrane protein 127 (TMEM127) [264], with TMEM127 interacting with WWP2 through a canonical binding motif (PPxY), so that the mMHCII–SteD–TMEM127–WWP2 complex is formed [263, 264] (Fig. 6). In this complex, WWP2 ubiquitylates mMHCII with K_63_-linked ubiquitin chains, targeting it for lysosomal degradation and resulting in reduced surface levels of mMHCII. Interestingly, SteD is also targeted for degradation through K_63_-linked ubiquitylation by WWP2 in a TMEM127-dependent manner and mMHCII depletion in the cell membrane is enhanced by SteD ubiquitylation [264]. Exactly how SteD ubiquitylation enhances mMHCII depletion is unclear [264], but host-mediated effector PTMs represent a common theme in effector activity. SteD, in complex with TMEM127 and WWP2, also facilitates degradation of the host protein CD97, with CD97 ubiquitylated by WWP2 at K_555_, targeting it for lysosomal degradation (Fig. 6) [265]. Overall, SteD fosters a reduction in mMHCII and CD97 at the cell surface of antigen presenting cells, reducing their interaction with T cells, dampening antigen presentation and reducing T-cell proliferation [263–265], permitting *Salmonella* to evade the adaptive immune response.

#### GogB: an F-box-like mimic

The exact molecular mechanism of GogB remains unknown. GogB contains an N-terminal canonical LRR domain, similar to that of SspH1, SspH2 and SlrP. Its C-terminal domain resembles an F-box-like domain, which mediates protein–protein interactions. Two host interacting partners, SKP1 and FBXO22, are described and via these proteins GogB inhibits NF-κB activation [266]. SKP1 represents a core component of the SKP1-Cul1-F-box (SCF) multi-protein ubiquitin ligase complex, in which F-box proteins, themselves bound to the adapter protein SKP1, recruit targets to the E3 ligase [267]. Whether the F-box-like domain in GogB, which is important for SKP1 interaction, directs SCF ligase activity towards new substrates is unclear. Instead, GogB somehow perturbs the ubiquitylation and degradation of IκBα, perhaps by abrogating the function of the SCF^FBOX22^ ligase complex, thereby preventing nuclear import of p65 and activation of NF-κB. *In vivo*, a *gogB* mutant induces greater inflammation and tissue damage as well as elevated inflammatory markers in the caecum [266].

#### SseF and SseG: tethering SCVs to the Golgi

SseF and SseG are two interdependent effectors encoded by the SPI-2 locus. They share approximately 35 % similarity at the amino acid level over their entire lengths and the same chaperone SscB [268]. Both effectors are integral membrane proteins [269, 270] with their N- and C-terminal regions facing the host cell cytoplasm [271–273]. Translocated SseF and SseG localize on the SCV membrane and *Salmonella *-induced tubules [274, 275]. Deleting one or both of the genes results in the same phenotype during *Salmonella* infection: (1) failure to form a microcolony and maintain SCVs close to the Golgi network in epithelial cells [269, 272, 276]; (2) formation of thinner, single membrane SIFs [274, 277] in epithelial cells and macrophages activated with IFN gamma; (3) failure to induce the aggregation of microtubules in epithelial cells [275]; (4) reduced ability to replicate in epithelial cells and macrophages [269, 274], and similar attenuation in a mouse infection model [269]; and (5) failure to form membrane tubules decorated with effectors but devoid of host proteins, i.e. LAMP1-negative tubules (LNTs) in the absence of SifA and SopD2 [278]. How SseF and SseG contribute to LNTs is unclear, but as they are verified integral membrane effectors localized to the SCV membrane, it is tempting to speculate that this requires their intrinsic ability to integrate into membranes.

Interaction between SseF and SseG [269] enables them to interact with the Golgi network-associated protein acyl-CoA binding domain containing 3 (ACBD3) [273], hence tethering the SCVs to the Golgi network to counteract movement away from the Golgi driven by microtubule motors and the effectors SteA and PipB2 [250, 276]. Lack of the interaction between SseF and SseG with ACBD3 also impairs intracellular bacterial replication [273], suggesting that proximity to the Golgi network might enable nutrient and/or membrane acquisition. Indeed, SseF is required for efficient access to endosomal cargo and nutrients in both epithelial and macrophage cells [277, 279], with intracellular bacteria lacking SseF entering a starvation stress response [280].

As well as ACBD3, SseF and SseG interact with Rab1A, inhibiting Rab1A’s interaction with its GEF, and Rab1A-mediated autophagy, thereby facilitating intracellular bacterial survival and replication [140]. Given that Rab1A is also involved in SIF formation in HeLa cells [281], the relative contribution of ACBD3 and Rab1A to the phenotypes attributed to SseF and SseG in both epithelial cells and macrophages requires further investigation. Notably, SseF has also been shown to interact with TIP60 [282] and junction plakoglobin [283], and SseG with desmoplakin and Caprin-1 [283] (Table 1), but the physiological importance of these interactions is unknown.

#### SifA and its role in membrane stability

SifA is an extensively studied ‘core’ effector with no confirmed enzymatic activity, yet it is very important for virulence [182]. SifA’s prototypical function is the stabilization of the SCV, preventing rupture and release of *Salmonella* into the cytosol [182]. As well as preserving the integrity of the vacuole, in epithelial cells SifA is required for the formation of SIFs, which are also referred to as *Salmonella *-induced tubules (SITs) [284]. These extended tubules are likely to enable nutrient acquisition for vacuolated bacteria [279]. Several other functions for SifA are described, including inhibition of lysosomal function through disruption of mannose 6-phosphate trafficking and Rab9 sequestration [285] as well as recruitment of the lysosomal tethering factor HOPS to the SCV [286]. Via its N-terminal domain (residues 1–136), SifA interacts with the C-terminal Pleckstrin Homology (PH) domain of host protein PLEKHM2 (SKIP) [183], mediating both SCV membrane stability and SIF formation through disruption of kinesin1 membrane localization [287]. Tethering of kinesin1 to the SCV also requires the host small GTPase Arl8b [288, 289] and *Salmonella* effector PipB2 [290] providing another example of effector co-operation.

SifA also interacts with the Rab7 effector PLEKHM1 via its N-terminal domain [263]. A complex consisting of SifA, PLEKHM1 and Rab7 results in recruitment of the HOPS complex to the SCV membrane [291]. Without this interaction, bacteria accumulate in large bag-like vacuoles, suggesting that the SifA–PLEKHM1 interaction enables the fission/fusion of late endosomes and lysosomes with the SCV to extend the vacuoles as the bacteria divide. While the N-terminal domain of SifA is required for PLEKHM1/2 interaction, its C-terminal domain contains a WxxxE motif indicative of GEFs [183]. Indeed, SifA demonstrates structural homology to the *Salmonella* effector SopE and other bacterial GEFs, suggesting it is a GEF mimic [292]. However, despite this, SifA has a non-homologous putative catalytic loop and no GEF activity has been demonstrated, despite interaction with GTPases Rab7 and RhoA [183, 293, 294].

Interestingly, there is a caspase-3 cleavage site in SifA, which is required for the proper localization of the N-terminal domain and C-terminal WxxxE motif-containing domain, as well as dissemination of bacteria to the liver following oral gavage [295]. This perhaps explains how SifA carries out multiple functions. Indeed, the C-terminal domain of SifA appears to independently recapitulate the activity of the protein in the absence of PLEKHM2 [296]. Furthermore, host-mediated prenylation at the C-terminal CAAX motif and S-acetylation of another C-terminal cysteine is required for SifA localization at the SCV membrane [297], emphasizing the importance of host-mediated PTMs to the function of this effector. Overall, the consensus is that, despite the WxxxE motif being important for SifA–PLEKHM2 interaction and a role for the putative catalytic loop in SIF formation and intracellular replication [298, 299], SifA functions as an adaptor.

#### SipA: an actin binding protein

SipA (SspA) is a SPI-1 effector and actin binding protein (ABP) with no recognized enzymatic activity. Instead, it facilitates bacterial entry through its C-terminal domain, which displaces host ABPs to prevent F-actin severing, and binds T-plastin to promote F-actin polymerization [39, 300]. The N-terminal domain of SipA mimics a cognate SNARE, mediating interaction with host Syntaxin8 to promote fusion with early endosomes [301]. Interestingly, similar to SifA, SipA is also a caspase-3 substrate, which releases the N-terminal domain from the actin-binding C-terminal domain [295].

### Enzymatic effectors with apparent non-enzymatic functions

With many effectors demonstrating diverse functions, it is not unexpected that some effectors show both enzymatic and non-enzymatic functions. Here we highlight some of these examples that probably require further investigation.

#### SseL and its interaction with OSBP

SseL interacts with, but does not appear to post-translationally modify, a lipid transporter called OSBP [302]. Indeed, a role for SseL in lipid regulation is supported by the observation that *sseL* mutant infected cells accumulate lipid droplets, which act as a store for cholesterol [303]. Furthermore, OSBP facilitates the transport of cholesterol between subcellular compartments [276] and appears important for *Salmonella* replication [302]. It is therefore interesting to speculate that SseL, in concert with SseJ, which also interacts with OSBP [184], co-opts OSBP to regulate cholesterol and lipid droplet formation. When both SseL and SseJ are absent, the *Salmonella* SCV is unstable, and this is phenocopied when the OSBP-interacting partner VAPA/B is knocked out [184]. Other host proteins might also be important, such as Niemann-Pick disease type C1 protein (NPC1), which is recruited to the SCV in an effector-dependent manner [24] and is known to regulate cholesterol storage. Overall, it remains to be determined how SseL regulates lipids during *Salmonella* infection, but together with SseJ, SseL is important for vacuole stability [24, 184].

#### SlrP and the chaperone ERdj3

SlrP interacts with the host chaperone protein, ERdj3, following partial localization to the ER [79]. However, no enzymatic activity of SlrP was required for the SlrP-mediated interference of ERdj3 binding to a denatured substrate [79] and a different effector tagging technique demonstrated that SlrP did not localize to the ER of epithelial cells in significant quantities [304].

#### SseK3 and its interaction with TRIM32

SseK3, but not SseK1 or SseK2, interacts with the host E3 ligase TRIM32 yet the significance of this remained unclear as TRIM32 was not identified as a substrate of SseK3, nor is it required for SseK3-mediated inhibition of NF-κB [133, 137]. Instead, TRIM32 ubiquitylates SseK3, targeting it for proteasomal degradation and therefore this interaction might represent a host-mediated response to infection [305].

### Poorly defined effectors

Since the discovery of the SPI-2 *Salmonella* system over 25 years ago [17], significant progress has been made in assigning the function and biochemical activity to a large proportion of its effectors (summarized in Table 1). Yet, we still know very little about several effectors. SteB, believed to be translocated by both the SPI-1 and SPI-2 injectisomes, is uncharacterized [306] and for CigR, which acts as an anti-virulence protein in competitive index experiments [148], it is not known if it functions within the host or the bacteria [307]. SifB is recruited to the SCV and shares a WxxxE motif with SifA [216]. Although SifB has been shown to co-purify with Rab13 and Rab10 in HeLa cells [24], it remains poorly characterized and has no described enzymatic activity. The crystal structure of SrfJ points to it resembling a glycoside hydrolase enzyme, yet very little is known about the proteins function [308].

For some effectors, host interaction partners have been described but with little further characterization. For example, PipB, recruits the ER-tethered protein PDZD8 to the SCV, and also interacts with the effector SifA [24], but the significance of these interactions is not clear. PipB2 interacts with several additional host proteins including kinesin light chain, KIF5B and more recently annexin A2, which also interacts with SopD2 [24, 244, 290]. However, how PipB2 impacts host PTMs and how these interaction partners support bacterial virulence requires further investigation. Finally, the ‘core’ effector SteA binds to phosphatidylinositol 4-phosphate (PI4P) in the SCV membrane, regulates vacuole membrane dynamics [309] and contributes to bacterial replication within vacuoles [310], yet further mechanistic insight is lacking. SteA also contributes to inhibition of NF-κB through its interaction with Cullin-1, a component of the SCF–E3 ligase complex that is also targeted by GogB [311].

### Conclusion and perspectives

The intensive, ongoing study of T3SS effector mechanisms has driven the discovery of diverse and novel biochemical modifications mediated by Gram-negative bacterial pathogens. With a cohort of over 40 T3SS effectors, *Salmonella* has offered a bounty of such discoveries, providing unprecedented insights into the mechanisms of bacterial virulence and host defence. The mediation or perturbation of PTMs by *Salmonella* T3SS effectors is a key virulence mechanism of the pathogen, often subverting host machineries by simply modulating pre-existing host molecules to establish a favourable replication niche and evade immune detection. Within a hostile intracellular milieu, *Salmonella* mediates precise and efficient cell-intrinsic immunity. The possible constraints on the quantity of effector molecules that can be translocated into the host cytosol would favour highly targeted enzymatic activity over purely protein–protein interactions. From enzymatic modification of host proteins at non-canonical sites to self-modification, host–enzymatic mimicry, novel effector–host modifications, effector activation by host–effector modification, or repurposing of host enzymes, *Salmonella* effectors act synergistically or antagonistically to facilitate pathogen survival. Such diversity of enzymatic activities underscores the complexity of co-evolution between the host and a highly successful intracellular pathogen over time and highlights how the rapid evolution of prokaryotic genomes can drive innovative protein biochemistry. Given the examples identified in this review and the current number of uncharacterized T3SS effectors, ongoing rigorous biochemical analysis is likely to reveal more effector-mediated PTMs, not only from *Salmonella* but multiple Gram-negative pathogens.

## References

[1] Kirk MD, Pires SM, Black RE, Caipo M, Crump JA (2015). World Health Organization estimates of the global and regional disease burden of 22 foodborne bacterial, protozoal, and viral diseases, 2010: a data synthesis. PLoS Med.

[2] Lee H, Yoon Y (2021). Etiological agents implicated in foodborne illness world wide. Food Sci Anim Resour.

[3] Pires SM, Desta BN, Mughini-Gras L, Mmbaga BT, Fayemi OE (2021). Burden of foodborne diseases: think global, act local. Curr Opin Food Sci.

[4] Chang Y-J, Chen Y-C, Chen N-W, Hsu Y-J, Chu H-H (2021). Changing antimicrobial resistance and epidemiology of non-typhoidal *Salmonella* infection in Taiwanese children. Front Microbiol.

[5] Holohan N, Wallat M, Hai Yen Luu T, Clark E, Truong DTQ (2022). Analysis of antimicrobial resistance in non-typhoidal *Salmonella* collected from pork retail outlets and slaughterhouses in Vietnam using whole genome sequencing. Front Vet Sci.

[6] Parisi A, Phuong TLT, Mather AE, Jombart T, Tuyen HT (2020). The role of animals as a source of antimicrobial resistant nontyphoidal *Salmonella* causing invasive and non-invasive human disease in Vietnam. Infect Genet Evol.

[7] Williamson DA, Lane CR, Easton M, Valcanis M, Strachan J (2018). Increasing antimicrobial resistance in nontyphoidal *Salmonella* isolates in Australia from 1979 to 2015. Antimicrob Agents Chemother.

[8] Coburn B, Sekirov I, Finlay BB (2007). Type III secretion systems and disease. Clin Microbiol Rev.

[9] Raffatellu M, Wilson RP, Chessa D, Andrews-Polymenis H, Tran QT (2005). SipA, SopA, SopB, SopD, and SopE2 contribute to *Salmonella enterica* serotype Typhimurium invasion of epithelial cells. Infect Immun.

[10] McGhie EJ, Brawn LC, Hume PJ, Humphreys D, Koronakis V (2009). *Salmonella* takes control: effector-driven manipulation of the host. Curr Opin Microbiol.

[11] Jennings E, Thurston TLM, Holden DW (2017). Salmonella SPI-2 type III secretion system effectors: molecular mechanisms and physiological consequences. Cell Host Microbe.

[12] Lou L, Zhang P, Piao R, Wang Y (2019). *Salmonella* pathogenicity island 1 (SPI-1) and its complex regulatory network. Front Cell Infect Microbiol.

[13] Macek B, Forchhammer K, Hardouin J, Weber-Ban E, Grangeasse C (2019). Protein post-translational modifications in bacteria. Nat Rev Microbiol.

[14] Chuh KN, Pratt MR (2015). Chemical methods for the proteome-wide identification of posttranslationally modified proteins. Curr Opin Chem Biol.

[15] Farley AR, Link AJ, Burgess RR, Deutscher MP (2009). Methods in Enzymology.

[16] Galán JE, Curtiss R (1989). Cloning and molecular characterization of genes whose products allow *Salmonella* typhimurium to penetrate tissue culture cells. Proc Natl Acad Sci.

[17] Hensel M, Shea JE, Gleeson C, Jones MD, Dalton E (1995). Simultaneous identification of bacterial virulence genes by negative selection. Science.

[18] Manning G, Whyte DB, Martinez R, Hunter T, Sudarsanam S (2002). The protein kinase complement of the human genome. Science.

[19] Hahn M, Covarrubias-Pinto A, Herhaus L, Satpathy S, Klann K (2021). SIK2 orchestrates actin-dependent host response upon *Salmonella* infection. Proc Natl Acad Sci.

[20] Imami K, Bhavsar AP, Yu H, Brown NF, Rogers LD (2013). Global impact of *Salmonella* pathogenicity island 2-secreted effectors on the host phosphoproteome. Mol Cell Proteomics.

[21] Heggie A, Cerny O, Holden DW (2021). SteC and the intracellular *Salmonella*-induced F-actin meshwork. Cell Microbiol.

[22] Poh J, Odendall C, Spanos A, Boyle C, Liu M (2008). SteC is a *Salmonella* kinase required for SPI-2-dependent F-actin remodelling. Cell Microbiol.

[23] Odendall C, Rolhion N, Förster A, Poh J, Lamont DJ (2012). The *Salmonella* kinase SteC targets the MAP kinase MEK to regulate the host actin cytoskeleton. Cell Host Microbe.

[24] Walch P, Selkrig J, Knodler LA, Rettel M, Stein F (2021). Global mapping of *Salmonella enterica*-host protein-protein interactions during infection. Cell Host Microbe.

[25] Park ER, Eblen ST, Catling AD (2007). MEK1 activation by PAK: a novel mechanism. Cell Signal.

[26] Kaniga K, Uralil J, Bliska JB, Galán JE (1996). A secreted protein tyrosine phosphatase with modular effector domains in the bacterial pathogen *Salmonella* typhimurlum. Mol Microbiol.

[27] Murli S, Watson RO, Galán JE (2001). Role of tyrosine kinases and the tyrosine phosphatase SptP in the interaction of *Salmonella* with host cells. Cell Microbiol.

[28] Pearl LH, Barford D (2002). Regulation of protein kinases in insulin, growth factor and Wnt signalling. Curr Opin Struct Biol.

[29] Stuckey JA, Schubert HL, Fauman EB, Zhang Z-Y, Dixon JE (1994). Crystal structure of *Yersinia* protein tyrosine phosphatase at 2.5 A and the complex with tungstate. Nature.

[30] Scheffzek K, Ahmadian MR, Wittinghofer A (1998). GTPase-activating proteins: helping hands to complement an active site. Trends Biochem Sci.

[31] Evdokimov AG, Tropea JE, Routzahn KM, Waugh DS (2002). Crystal structure of the *Yersinia pestis* GTPase activator YopE. Protein Sci.

[32] Würtele M, Renault L, Barbieri JT, Wittinghofer A, Wolf E (2001). Structure of the ExoS GTPase activating domain. FEBS Lett.

[33] Chen L-M, Hobbie S, Galán JE (1996). Requirement of CDC42 for *Salmonella* -induced cytoskeletal and nuclear responses. Science.

[34] Ridley AJ, Paterson HF, Johnston CL, Diekmann D, Hall A (1992). The small GTP-binding protein rac regulates growth factor-induced membrane ruffling. Cell.

[35] Stebbins CE, Galán JE (2000). Modulation of host signaling by a bacterial mimic: structure of the *Salmonella* effector SptP bound to Rac1. Mol Cell.

[36] Fu Y, Galán JE (1999). A *Salmonella* protein antagonizes Rac-1 and Cdc42 to mediate host-cell recovery after bacterial invasion. Nature.

[37] Hardt W-D, Chen L-M, Schuebel KE, Bustelo XR, Galán JE (1998). *S. typhimurium* encodes an activator of Rho GTPases that induces membrane ruffling and nuclear responses in host cells. Cell.

[38] Kubori T, Galán JE (2003). Temporal regulation of *Salmonella* virulence effector function by proteasome-dependent protein degradation. Cell.

[39] Lhocine N, Arena ET, Bomme P, Ubelmann F, Prévost M-C (2015). Apical invasion of intestinal epithelial cells by *Salmonella* typhimurium requires villin to remodel the brush border actin cytoskeleton. Cell Host & Microbe.

[40] Humphreys D, Hume PJ, Koronakis V (2009). The *Salmonella* effector SptP dephosphorylates host AAA+ ATPase VCP to promote development of its intracellular replicative niche. Cell Host Microbe.

[41] Choi HW, Brooking-Dixon R, Neupane S, Lee C-J, Miao EA (2013). *Salmonella* typhimurium impedes innate immunity with a mast-cell-suppressing protein tyrosine phosphatase, SptP. Immunity.

[42] Zhu Y, Li H, Long C, Hu L, Xu H (2007). Structural insights into the enzymatic mechanism of the pathogenic MAPK phosphothreonine lyase. Mol Cell.

[43] Johnson R, Byrne A, Berger CN, Klemm E, Crepin VF (2017). The type III secretion system effector SptP of *Salmonella enterica* serovar Typhi. J Bacteriol.

[44] Chen L-M, Bagrodia S, Cerione RA, Galán JE (1999). Requirement of p21-activated kinase (PAK) for *Salmonella* typhimurium-induced nuclear responses. J Exp Med.

[45] Lin SL, Le TX, Cowen DS (2003). SptP, a *Salmonella* typhimurium type III-secreted protein, inhibits the mitogen-activated protein kinase pathway by inhibiting Raf activation. Cell Microbiol.

[46] Norris FA, Wilson MP, Wallis TS, Galyov EE, Majerus PW (1998). SopB, a protein required for virulence of *Salmonella* dublin, is an inositol phosphate phosphatase. Proc Natl Acad Sci.

[47] Patel JC, Galán JE (2006). Differential activation and function of Rho GTPases during *Salmonella*-host cell interactions. J Cell Biol.

[48] Patel JC, Hueffer K, Lam TT, Galán JE (2009). Diversification of a *Salmonella* virulence protein function by ubiquitin-dependent differential localization. Cell.

[49] Steele-Mortimer O, Knodler LA, Marcus SL, Scheid MP, Goh B (2000). Activation of Akt/protein kinase B in epithelial cells by the *Salmonella* typhimurium effector sigD. J Biol Chem.

[50] Mallo GV, Espina M, Smith AC, Terebiznik MR, Alemán A (2008). SopB promotes phosphatidylinositol 3-phosphate formation on *Salmonella* vacuoles by recruiting Rab5 and Vps34. J Cell Biol.

[51] Hänisch J, Kölm R, Wozniczka M, Bumann D, Rottner K (2011). Activation of a RhoA/Myosin II-dependent but Arp2/3 complex-independent pathway facilitates *Salmonella* invasion. Cell Host & Microbe.

[52] Burkinshaw BJ, Prehna G, Worrall LJ, Strynadka NCJ (2012). Structure of *Salmonella* effector protein SopB N-terminal domain in complex with host Rho GTPase Cdc42. J Biol Chem.

[53] Zhao S, Xu Q, Cui Y, Yao S, Jin S (2023). *Salmonella* effector SopB reorganizes cytoskeletal vimentin to maintain replication vacuoles for efficient infection. Nat Commun.

[54] Mazurkiewicz P, Thomas J, Thompson JA, Liu M, Arbibe L (2008). SpvC is a *Salmonella* effector with phosphothreonine lyase activity on host mitogen-activated protein kinases. Mol Microbiol.

[55] Smith GK, Ke Z, Hengge AC, Xu D, Xie D (2009). Active-site dynamics of SpvC virulence factor from *Salmonella* typhimurium and density functional theory study of phosphothreonine lyase catalysis. J Phys Chem B.

[56] Li H, Xu H, Zhou Y, Zhang J, Long C (2007). The phosphothreonine lyase activity of a bacterial type III effector family. Science.

[57] Miki T, Akiba K, Iguchi M, Danbara H, Okada N (2011). The *Chromobacterium violaceum* type III effector CopE, a guanine nucleotide exchange factor for Rac1 and Cdc42, is involved in bacterial invasion of epithelial cells and pathogenesis. Mol Microbiol.

[58] Zhang X, Liu W, Li Y, Li G, Xu JR (2017). Expression of HopAI interferes with MAP kinase signalling in *Magnaporthe oryzae*. Environ Microbiol.

[59] Haneda T, Ishii Y, Shimizu H, Ohshima K, Iida N (2012). *Salmonella* type III effector SpvC, a phosphothreonine lyase, contributes to reduction in inflammatory response during intestinal phase of infection. Cell Microbiol.

[60] Hershko A, Ciechanover A (1998). The ubiquitin system. Annu Rev Biochem.

[61] Lilienbaum A (2013). Relationship between the proteasomal system and autophagy. Int J Biochem Mol Biol.

[62] Narayanan LA, Edelmann MJ (2014). Ubiquitination as an efficient molecular strategy employed in *Salmonella* infection. Front Immunol.

[63] Schreiber A, Peter M (2014). Substrate recognition in selective autophagy and the ubiquitin-proteasome system. Biochim Biophys Acta.

[64] Shaid S, Brandts CH, Serve H, Dikic I (2013). Ubiquitination and selective autophagy. Cell Death Differ.

[65] Chen R-H, Chen Y-H, Huang T-Y (2019). Ubiquitin-mediated regulation of autophagy. J Biomed Sci.

[66] Otten EG, Werner E, Crespillo-Casado A, Boyle KB, Dharamdasani V (2021). Ubiquitylation of lipopolysaccharide by RNF213 during bacterial infection. Nature.

[67] French ME, Koehler CF, Hunter T (2021). Emerging functions of branched ubiquitin chains. Cell Discov.

[68] Fiskin E, Bionda T, Dikic I, Behrends C (2016). Global analysis of host and bacterial ubiquitinome in response to *Salmonella* Typhimurium infection. Mol Cell.

[69] Wood MW, Jones MA, Watson PR, Siber AM, McCormick BA (2000). The secreted effector protein of *Salmonella* dublin, SopA, is translocated into eukaryotic cells and influences the induction of enteritis. Cell Microbiol.

[70] Zhang S, Santos RL, Tsolis RM, Stender S, Hardt W-D (2002). The *Salmonella enterica* serotype Typhimurium effector proteins SipA, SopA, SopB, SopD, and SopE2 act in concert to induce diarrhea in calves. Infect Immun.

[71] Zhang Y, Higashide W, Dai S, Sherman DM, Zhou D (2005). Recognition and ubiquitination of *Salmonella* type III effector SopA by a ubiquitin E3 ligase, HsRMA1. J Biol Chem.

[72] Zhang Y, Higashide WM, McCormick BA, Chen J, Zhou D (2006). The inflammation-associated *Salmonella* SopA is a HECT-like E3 ubiquitin ligase. Mol Microbiol.

[73] Kamanova J, Sun H, Lara-Tejero M, Galán JE (2016). The *Salmonella* effector protein SopA modulates innate immune responses by targeting TRIM E3 ligase family members. PLoS Pathog.

[74] Fiskin E, Bhogaraju S, Herhaus L, Kalayil S, Hahn M (2017). Structural basis for the recognition and degradation of host TRIM proteins by *Salmonella* effector SopA. Nat Commun.

[75] Lin DY, Diao J, Zhou D, Chen J (2011). Biochemical and structural studies of a HECT-like ubiquitin ligase from *Escherichia coli* O157:H7. J Biol Chem.

[76] Lin DY, Diao J, Chen J (2012). Crystal structures of two bacterial HECT-like E3 ligases in complex with a human E2 reveal atomic details of pathogen-host interactions. Proc Natl Acad Sci.

[77] Piscatelli H, Kotkar SA, McBee ME, Muthupalani S, Schauer DB (2011). The EHEC type III effector NleL is an E3 ubiquitin ligase that modulates pedestal formation. PLoS One.

[78] Quezada CM, Hicks SW, Galán JE, Stebbins CE (2009). A family of *Salmonella* virulence factors functions as a distinct class of autoregulated E3 ubiquitin ligases. Proc Natl Acad Sci.

[79] Bernal-Bayard J, Cardenal-Muñoz E, Ramos-Morales F (2010). The *Salmonella* type III secretion effector, salmonella leucine-rich repeat protein (SlrP), targets the human chaperone ERdj3. J Biol Chem.

[80] Bernal-Bayard J, Ramos-Morales F (2009). *Salmonella* type III secretion effector SlrP is an E3 ubiquitin ligase for mammalian thioredoxin. J Biol Chem.

[81] Bullones-Bolaños A, Araujo-Garrido JL, Fernández-García J, Romero F, Bernal-Bayard J (2022). SNRPD2 is a novel substrate for the ubiquitin ligase activity of the *Salmonella* type III secretion effector SlrP. Biology.

[82] Cook M, Delbecq SP, Schweppe TP, Guttman M, Klevit RE (2019). The ubiquitin ligase SspH1 from *Salmonella* uses a modular and dynamic E3 domain to catalyze substrate ubiquitylation. J Biol Chem.

[83] Zouhir S, Bernal-Bayard J, Cordero-Alba M, Cardenal-Muñoz E, Guimaraes B (2014). The structure of the Slrp-Trx1 complex sheds light on the autoinhibition mechanism of the type III secretion system effectors of the NEL family. Biochem J.

[84] Holmgren A (1985). Thioredoxin. Annu Rev Biochem.

[85] Matthews JR, Wakasugi N, Virelizier JL, Yodoi J, Hay RT (1992). Thioredoxin regulates the DNA binding activity of NF-kappa B by reduction of a disulphide bond involving cysteine 62. Nucleic Acids Res.

[86] Muri J, Thut H, Feng Q, Kopf M (2020). Thioredoxin-1 distinctly promotes NF-κB target DNA binding and NLRP3 inflammasome activation independently of Txnip. Elife.

[87] Halici S, Zenk SF, Jantsch J, Hensel M (2008). Functional analysis of the *Salmonella* pathogenicity island 2-mediated inhibition of antigen presentation in dendritic cells. Infect Immun.

[88] Miao EA, Scherer CA, Tsolis RM, Kingsley RA, Adams LG (1999). *Salmonella* typhimurium leucine-rich repeat proteins are targeted to the SPI1 and SPI2 type III secretion systems. Mol Microbiol.

[89] Rao S, Schieber AMP, O’Connor CP, Leblanc M, Michel D (2017). Pathogen-mediated inhibition of anorexia promotes host survival and transmission. Cell.

[90] De Meyer M, Fijalkowski I, Jonckheere V, De Sutter D, Eyckerman S (2021). Capturing *Salmonella* SspH2 host targets in virus-like particles. Front Med.

[91] Bullones-Bolaños A, Bernal-Bayard J, Ramos-Morales F (2022). The NEL family of bacterial E3 ubiquitin ligases. Int J Mol Sci.

[92] Haraga A, Miller SI (2003). A *Salmonella enterica* serovar typhimurium translocated leucine-rich repeat effector protein inhibits NF-kappa B-dependent gene expression. Infect Immun.

[93] Keszei AFA, Tang X, McCormick C, Zeqiraj E, Rohde JR (2014). Structure of an SspH1-PKN1 complex reveals the basis for host substrate recognition and mechanism of activation for a bacterial E3 ubiquitin ligase. Mol Cell Biol.

[94] Haraga A, Miller SI (2006). A *Salmonella* type III secretion effector interacts with the mammalian serine/threonine protein kinase PKN1. Cell Microbiol.

[95] Metzger E, Imhof A, Patel D, Kahl P, Hoffmeyer K (2010). Phosphorylation of histone H3T6 by PKCβI controls demethylation at histone H3K4. Nature.

[96] Metzger E, Müller JM, Ferrari S, Buettner R, Schüle R (2003). A novel inducible transactivation domain in the androgen receptor: implications for PRK in prostate cancer. EMBO J.

[97] Metzger E, Yin N, Wissmann M, Kunowska N, Fischer K (2008). Phosphorylation of histone H3 at threonine 11 establishes a novel chromatin mark for transcriptional regulation. Nat Cell Biol.

[98] Rohde JR, Breitkreutz A, Chenal A, Sansonetti PJ, Parsot C (2007). Type III secretion effectors of the IpaH family are E3 ubiquitin ligases. Cell Host Microbe.

[99] Kato Jr T, Gotoh Y, Hoffmann A, Ono Y (2008). Negative regulation of constitutive NF-κB and JNK signaling by PKN1-mediated phosphorylation of TRAF1. Genes Cells.

[100] Bhavsar AP, Brown NF, Stoepel J, Wiermer M, Martin DDO (2013). The *Salmonella* type III effector SspH2 specifically exploits the NLR co-chaperone activity of SGT1 to subvert immunity. PLoS Pathog.

[101] da Silva Correia J, Miranda Y, Leonard N, Ulevitch R (2007). SGT1 is essential for Nod1 activation. Proc Natl Acad Sci.

[102] Hong T-J, Hahn J-S (2016). Application of SGT1-Hsp90 chaperone complex for soluble expression of NOD1 LRR domain in E. coli. Biochem Biophys Res Commun.

[103] Sirard J-C, Vignal C, Dessein R, Chamaillard M (2007). Nod-like receptors: cytosolic watchdogs for immunity against pathogens. PLoS Pathog.

[104] Wilmanski JM, Petnicki-Ocwieja T, Kobayashi KS (2008). NLR proteins: integral members of innate immunity and mediators of inflammatory diseases. J Leukoc Biol.

[105] Ashida H, Sasakawa C (2017). Bacterial E3 ligase effectors exploit host ubiquitin systems. Curr Opin Microbiol.

[106] Dranenko NO, Tutukina MN, Gelfand MS, Kondrashov FA, Bochkareva OO (2022). Chromosome-encoded IpaH ubiquitin ligases indicate non-human enteroinvasive *Escherichia*. Sci Rep.

[107] Wandel MP, Pathe C, Werner EI, Ellison CJ, Boyle KB (2017). GBPs inhibit motility of *Shigella flexneri* but are targeted for degradation by the bacterial ubiquitin ligase IpaH9.8. Cell Host Microbe.

[108] Pruneda JN, Durkin CH, Geurink PP, Ovaa H, Santhanam B (2016). The molecular basis for ubiquitin and ubiquitin-like specificities in bacterial effector proteases. Mol Cell.

[109] Rytkönen A, Poh J, Garmendia J, Boyle C, Thompson A (2007). SseL, a *Salmonella* deubiquitinase required for macrophage killing and virulence. Proc Natl Acad Sci.

[110] Geng S, Wang Y, Xue Y, Wang H, Cai Y (2019). The SseL protein inhibits the intracellular NF-κB pathway to enhance the virulence of *Salmonella* Pullorum in a chicken model. Microb Pathog.

[111] Le Negrate G, Faustin B, Welsh K, Loeffler M, Krajewska M (2008). *Salmonella* secreted factor L deubiquitinase of *Salmonella* typhimurium inhibits NF-kappaB, suppresses IkappaBalpha ubiquitination and modulates innate immune responses. J Immunol.

[112] Mesquita FS, Holden DW, Rolhion N, Gorvel J-P (2013). Lack of effect of the *Salmonella* deubiquitinase SseL on the NF-κB pathway. PLoS One.

[113] Wan F, Anderson DE, Barnitz RA, Snow A, Bidere N (2007). Ribosomal protein S3: a KH domain subunit in NF-kappaB complexes that mediates selective gene regulation. Cell.

[114] Wan F, Lenardo MJ (2009). Specification of DNA binding activity of NF-kappaB proteins. Cold Spring Harb Perspect Biol.

[115] Wu M, El Qaidi S, Hardwidge PR (2018). SseL deubiquitinates RPS3 to inhibit its nuclear translocation. Pathogens.

[116] Le Negrate G, Krieg A, Faustin B, Loeffler M, Godzik A (2008). *Chla* Dub1 of *Chlamydia trachomatis* suppresses NF-κB activation and inhibits IκBα ubiquitination and degradation. Cell Microbiol.

[117] Misaghi S, Balsara ZR, Catic A, Spooner E, Ploegh HL (2006). *Chlamydia trachomatis* -derived deubiquitinating enzymes in mammalian cells during infection. Mol Microbiol.

[118] Catic A, Misaghi S, Korbel GA, Ploegh HL, Carr J (2007). ElaD, a deubiquitinating protease expressed by *E. coli*. PLoS One.

[119] Mesquita FS, Thomas M, Sachse M, Santos AJM, Figueira R (2012). The *Salmonella* deubiquitinase SseL inhibits selective autophagy of cytosolic aggregates. PLoS Pathog.

[120] Li S, Zhang L, Yao Q, Li L, Dong N (2013). Pathogen blocks host death receptor signalling by arginine GlcNAcylation of death domains. Nature.

[121] Pearson JS, Giogha C, Ong SY, Kennedy CL, Kelly M (2013). A type III effector antagonizes death receptor signalling during bacterial gut infection. Nature.

[122] Scott NE, Giogha C, Pollock GL, Kennedy CL, Webb AI (2017). The bacterial arginine glycosyltransferase effector NleB preferentially modifies Fas-associated death domain protein (FADD). J Biol Chem.

[123] Brown NF, Coombes BK, Bishop JL, Wickham ME, Lowden MJ (2011). *Salmonella* phage ST64B encodes a member of the SseK/NleB effector family. PLoS One.

[124] Kujat Choy SL, Boyle EC, Gal-Mor O, Goode DL, Valdez Y (2004). SseK1 and SseK2 are novel translocated proteins of *Salmonella enterica* serovar typhimurium. Infect Immun.

[125] Park JB, Kim YH, Yoo Y, Kim J, Jun S-H (2018). Structural basis for arginine glycosylation of host substrates by bacterial effector proteins. Nat Commun.

[126] Esposito D, Günster RA, Martino L, El Omari K, Wagner A (2018). Structural basis for the glycosyltransferase activity of the *Salmonella* effector SseK3. J Biol Chem.

[127] Ding J, Pan X, Du L, Yao Q, Xue J (2019). Structural and functional insights into host death domains inactivation by the bacterial arginine GlcNAcyltransferase effector. Molecular Cell.

[128] Ardèvol A, Iglesias-Fernández J, Rojas-Cervellera V, Rovira C (2016). The reaction mechanism of retaining glycosyltransferases. Biochem Soc Trans.

[129] Albesa-Jové D, Sainz-Polo MÁ, Marina A, Guerin ME (2017). Structural snapshots of α-1,3-galactosyltransferase with native substrates: insight into the catalytic mechanism of retaining glycosyltransferases. Angew Chem Int Ed Engl.

[130] Ardèvol A, Rovira C (2011). The molecular mechanism of enzymatic glycosyl transfer with retention of configuration: evidence for a short-lived oxocarbenium-like species. Angew Chem Int Ed Engl.

[131] Schuman B, Evans SV, Fyles TM (2013). Geometric attributes of retaining glycosyltransferase enzymes favor an orthogonal mechanism. PLoS One.

[132] Lazarus MB, Nam Y, Jiang J, Sliz P, Walker S (2011). Structure of human O-GlcNAc transferase and its complex with a peptide substrate. Nature.

[133] Günster RA, Matthews SA, Holden DW, Thurston TLM, Bäumler AJ (2017). SseK1 and SseK3 type III secretion system effectors inhibit NF-κB signaling and necroptotic cell death in *Salmonella*-infected macrophages. Infect Immun.

[134] Pan M, Li S, Li X, Shao F, Liu L (2014). Synthesis of and specific antibody generation for glycopeptides with arginine *N* -GlcNAcylation. Angew Chem Int Ed.

[135] El Qaidi S, Chen K, Halim A, Siukstaite L, Rueter C (2017). NleB/SseK effectors from *Citrobacter rodentium*, *Escherichia coli*, and *Salmonella enterica* display distinct differences in host substrate specificity. J Biol Chemist.

[136] Newson JPM, Scott NE, Yeuk Wah Chung I, Wong Fok Lung T, Giogha C (2019). *Salmonella* effectors SseK1 and SseK3 target death domain proteins in the TNF and TRAIL signaling pathways. Mol Cell Proteomics.

[137] Yang Z, Soderholm A, Lung TWF, Giogha C, Hill MM (2015). SseK3 is a *Salmonella* effector that binds TRIM32 and modulates the host’s NF-κB signalling activity. PLoS One.

[138] Gan J, Scott NE, Newson JPM, Wibawa RR, Wong Fok Lung T (2020). The *Salmonella* effector SseK3 targets small Rab GTPases. Front Cell Infect Microbiol.

[139] Meng K, Zhuang X, Peng T, Hu S, Yang J (2020). Arginine GlcNAcylation of rab small GTPases by the pathogen *Salmonella* Typhimurium. Commun Biol.

[140] Feng Z-Z, Jiang A-J, Mao A-W, Feng Y, Wang W (2018). The *Salmonella* effectors SseF and SseG inhibit Rab1A-mediated autophagy to facilitate intracellular bacterial survival and replication. J Biol Chem.

[141] Mukherjee K, Parashuraman S, Raje M, Mukhopadhyay A (2001). SopE acts as an Rab5-specific nucleotide exchange factor and recruits non-prenylated Rab5 on *Salmonella*-containing phagosomes to promote fusion with early endosomes. J Biol Chem.

[142] García-García A, Hicks T, El Qaidi S, Zhu C, Hardwidge PR (2021). NleB/SseK-catalyzed arginine-glycosylation and enteropathogen virulence are finely tuned by a single variable position contiguous to the catalytic machinery. Chem Sci.

[143] Giogha C, Scott NE, Wong Fok Lung T, Pollock GL, Harper M (2021). NleB2 from enteropathogenic *Escherichia coli* is a novel arginine-glucose transferase effector. PLOS Pathog.

[144] Xue J, Huang Y, Zhang H, Hu J, Pan X (2021). Arginine GlcNAcylation and activity regulation of PhoP by a type III secretion system effector in *Salmonella*. Front Microbiol.

[145] Xue J, Pan X, Peng T, Duan M, Du L (2020). Auto arginine-GlcNAcylation is crucial for bacterial pathogens in regulating host cell death. Front Cell Infect Microbiol.

[146] Baisón-Olmo F, Galindo-Moreno M, Ramos-Morales F (2015). Host cell type-dependent translocation and PhoP-mediated positive regulation of the effector SseK1 of *Salmonella enterica*. Front Microbiol.

[147] Buckner MMC, Croxen M, Arena ET, Finlay BB (2011). A comprehensive study of the contribution of *Salmonella enterica* serovar Typhimurium SPI2 effectors to bacterial colonization, survival, and replication in typhoid fever, macrophage, and epithelial cell infection models. Virulence.

[148] Kidwai AS, Mushamiri I, Niemann GS, Brown RN, Adkins JN (2013). Diverse secreted effectors are required for *Salmonella* persistence in a mouse infection model. PLoS One.

[149] Yang Y, Yu C, Ding K, Zhang C, Liao C (2018). Role of the sseK1 gene in the pathogenicity of *Salmonella enterica* serovar enteritidis in vitro and in vivo. Microb Pathog.

[150] Hoch NC, Polo LM (2020). ADP-ribosylation: from molecular mechanisms to human disease. Genet Mol Biol.

[151] Caldwell AL, Gulig PA (1991). The *Salmonella* typhimurium virulence plasmid encodes a positive regulator of a plasmid-encoded virulence gene. J Bacteriol.

[152] Lesnick ML, Reiner NE, Fierer J, Guiney DG (2001). The *Salmonella* spvB virulence gene encodes an enzyme that ADP-ribosylates actin and destabilizes the cytoskeleton of eukaryotic cells. Mol Microbiol.

[153] Otto H, Tezcan-Merdol D, Girisch R, Haag F, Rhen M (2000). The spvB gene-product of the *Salmonella enterica* virulence plasmid is a mono(ADP-ribosyl)transferase. Mol Microbiol.

[154] Margarit SM, Davidson W, Frego L, Stebbins CE (2006). A steric antagonism of actin polymerization by a *Salmonella* virulence protein. Structure.

[155] Tezcan-Merdol D, Nyman T, Lindberg U, Haag F, Koch-Nolte F (2001). Actin is ADP-ribosylated by the *Salmonella enterica* virulence-associated protein SpvB. Mol Microbiol.

[156] Hochmann H, Pust S, von Figura G, Aktories K, Barth H (2006). *Salmonella enterica* SpvB ADP-ribosylates actin at position arginine-177-characterization of the catalytic domain within the SpvB protein and a comparison to binary clostridial actin-ADP-ribosylating toxins. Biochemistry.

[157] Schüler H, Nyåkern M, Schutt CE, Lindberg U, Karlsson R (2000). Mutational analysis of arginine 177 in the nucleotide binding site of β-actin. Eur J Biochem.

[158] Birmingham CL, Jiang X, Ohlson MB, Miller SI, Brumell JH (2005). *Salmonella* -induced filament formation is a dynamic phenotype induced by rapidly replicating *Salmonella enterica* serovar Typhimurium in epithelial cells. Infect Immun.

[159] Browne SH, Lesnick ML, Guiney DG (2002). Genetic requirements for *Salmonella*-induced cytopathology in human monocyte-derived macrophages. Infect Immun.

[160] Libby SJ, Lesnick M, Hasegawa P, Weidenhammer E, Guiney DG (2000). The *Salmonella* virulence plasmid spv genes are required for cytopathology in human monocyte-derived macrophages. Cell Microbiol.

[161] Eulalio A, Fröhlich KS, Mano M, Giacca M, Vogel J (2011). A candidate approach implicates the secreted *Salmonella* effector protein SpvB in P-body disassembly. PLoS One.

[162] Sun L, Yang S, Deng Q, Dong K, Li Y (2020). *Salmonella* effector SpvB disrupts intestinal epithelial barrier integrity for bacterial translocation. Front Cell Infect Microbiol.

[163] Yang S, Deng Q, Sun L, Dong K, Li Y (2019). *Salmonella* effector SpvB interferes with intracellular iron homeostasis *via* regulation of transcription factor NRF2. FASEB J.

[164] Yang S, Deng Q, Sun L, Zhu Y, Dong K (2021). *Salmonella* effector SpvB inhibits NF-κB activity via KEAP1-mediated downregulation of IKKβ. Front Cell Infect Microbiol.

[165] Basit A, Tahir H, Haider Z, Tariq H, Ullah A (2022). CRISPR/Cas9-based deletion of SpvB gene from *Salmonella gallinarum* leads to loss of virulence in chicken. Front Bioeng Biotechnol.

[166] Käppeli R, Kaiser P, Stecher B, Hardt WD (2011). Roles of spvB and spvC in *S*. Typhimurium colitis via the alternative pathway. Int J Med Microbiol.

[167] Matsui H, Bacot CM, Garlington WA, Doyle TJ, Roberts S (2001). Virulence plasmid-borne *spvB* and *spvC* genes can replace the 90-Kilobase plasmid in conferring virulence to *Salmonella enterica* serovar Typhimurium in subcutaneously inoculated mice. J Bacteriol.

[168] Cheng S, Wang L, Liu Q, Qi L, Yu K (2017). Identification of a novel *Salmonella* type III effector by quantitative secretome profiling. Mol Cell Proteomics.

[169] Xu Y, Zhou P, Cheng S, Lu Q, Nowak K (2019). A bacterial effector reveals the V-ATPase-ATG16L1 axis that initiates xenophagy. Cell.

[170] Xu Y, Cheng S, Zeng H, Zhou P, Ma Y (2022). ARF GTPases activate *Salmonella* effector SopF to ADP-ribosylate host V-ATPase and inhibit endomembrane damage-induced autophagy. Nat Struct Mol Biol.

[171] Lau N, Haeberle AL, O’Keeffe BJ, Latomanski EA, Celli J (2019). SopF, a phosphoinositide binding effector, promotes the stability of the nascent *Salmonella*-containing vacuole. PLoS Pathog.

[172] Ohlson MB, Fluhr K, Birmingham CL, Brumell JH, Miller SI (2005). SseJ deacylase activity by *Salmonella enterica* serovar Typhimurium promotes virulence in mice. Infect Immun.

[173] Upton C, Buckley JT (1995). A new family of lipolytic enzymes?. Trends Biochem Sci.

[174] Lossi NS, Rolhion N, Magee AI, Boyle C, Holden DW (2008). The *Salmonella* SPI-2 effector SseJ exhibits eukaryotic activator-dependent phospholipase A and glycerophospholipid: cholesterol acyltransferase activity. Microbiology.

[175] Nawabi P, Catron DM, Haldar K (2008). Esterification of cholesterol by a type III secretion effector during intracellular *Salmonella* infection. Mol Microbiol.

[176] Christen M, Coye LH, Hontz JS, LaRock DL, Pfuetzner RA (2009). Activation of a bacterial virulence protein by the GTPase RhoA. Sci Signal.

[177] LaRock DL, Brzovic PS, Levin I, Blanc MP, Miller SI (2012). A *Salmonella* typhimurium-translocated glycerophospholipid:cholesterol acyltransferase promotes virulence by binding to the RhoA protein switch regions. J Biol Chem.

[178] Freeman JA, Ohl ME, Miller SI (2003). The *Salmonella enterica* serovar Typhimurium translocated effectors SseJ and SifB are targeted to the *Salmonella* -containing vacuole. Infect Immun.

[179] Ruiz-Albert J, Yu XJ, Beuzón CR, Blakey AN, Galyov EE (2002). Complementary activities of SseJ and SifA regulate dynamics of the *Salmonella* typhimurium vacuolar membrane. Mol Microbiol.

[180] Brumell JH, Goosney DL, Finlay BB (2002). SifA, a type III secreted effector of *Salmonella* typhimurium, directs *Salmonella*-induced filament (Sif) formation along microtubules. Traffic.

[181] Kolodziejek AM, Miller SI (2015). *Salmonella* modulation of the phagosome membrane, role of SseJ. Cell Microbiol.

[182] Beuzon CR (2000). *Salmonella* maintains the integrity of its intracellular vacuole through the action of SifA. Embo j.

[183] Ohlson MB, Huang Z, Alto NM, Blanc M-P, Dixon JE (2008). Structure and function of *Salmonella* SifA indicate that its interactions with SKIP, SseJ, and RhoA family GTPases induce endosomal tubulation. Cell Host Microbe.

[184] Kolodziejek AM, Altura MA, Fan J, Petersen EM, Cook M (2019). *Salmonella* translocated effectors recruit OSBP1 to the phagosome to promote vacuolar membrane integrity. Cell Reports.

[185] Greene AR, Owen KA, Casanova JE (2021). *Salmonella* Typhimurium manipulates macrophage cholesterol homeostasis through the SseJ-mediated suppression of the host cholesterol transport protein ABCA1. Cell Microbiol.

[186] Ma KW, Ma W (2016). YopJ family effectors promote bacterial infection through a unique acetyltransferase activity. Microbiol Mol Biol Rev.

[187] Mittal R, Peak-Chew SY, Sade RS, Vallis Y, McMahon HT (2010). The acetyltransferase activity of the bacterial toxin YopJ of *Yersinia* is activated by eukaryotic host cell inositol hexakisphosphate. J Biol Chem.

[188] Wu S, Ye Z, Liu X, Zhao Y, Xia Y (2010). *Salmonella* typhimurium infection increases p53 acetylation in intestinal epithelial cells. Am J Physiol Gastrointest Liver Physiol.

[189] Collier-Hyams LS, Zeng H, Sun J, Tomlinson AD, Bao ZQ (2002). Cutting Edge: *Salmonella* AvrA effector inhibits the key proinflammatory, anti-apoptotic NF-κB pathway. J Immunol.

[190] Du F, Galán JE, Stebbins CE (2009). Selective inhibition of type III secretion activated signaling by the *Salmonella* effector AvrA. PLoS Pathog.

[191] Jones RM, Wu H, Wentworth C, Luo L, Collier-Hyams L (2008). *Salmonella* AvrA coordinates suppression of host immune and apoptotic defenses via JNK pathway blockade. Cell Host Microbe.

[192] Labriola JM, Zhou Y, Nagar B (2018). Structural analysis of the bacterial effector AvrA identifies a critical helix involved in substrate recognition. Biochemistry.

[193] Jiao Y, Zhang Y, Lin Z, Lu R, Xia Y (2020). *Salmonella* enteritidis effector AvrA suppresses autophagy by reducing beclin-1 Protein. Front Immunol.

[194] Liao AP, Petrof EO, Kuppireddi S, Zhao Y, Xia Y (2008). *Salmonella* type III effector AvrA stabilizes cell tight junctions to inhibit inflammation in intestinal epithelial cells. PLoS One.

[195] Liu X, Lu R, Wu S, Zhang Y-G, Xia Y (2012). Wnt2 inhibits enteric bacterial-induced inflammation in intestinal epithelial cells. Inflamm Bowel Dis.

[196] Lu R, Liu X, Wu S, Xia Y, Zhang Y-G (2012). Consistent activation of the β-catenin pathway by *Salmonella* type-three secretion effector protein AvrA in chronically infected intestine. Am J Physiol Gastrointest Liver Physiol.

[197] Lu R, Wu S, Zhang Y, Xia Y, Liu X (2014). Enteric bacterial protein AvrA promotes colonic tumorigenesis and activates colonic beta-catenin signaling pathway. Oncogenesis.

[198] Lu R, Wu S, Zhang Y, Xia Y, Zhou Z (2016). *Salmonella* protein AvrA activates the STAT3 signaling pathway in colon cancer. Neoplasia.

[199] Ye Z, Petrof EO, Boone D, Claud EC, Sun J (2007). *Salmonella* effector AvrA regulation of colonic epithelial cell inflammation by deubiquitination. Am J Pathol.

[200] Yin C, Liu Z, Xian H, Jiao Y, Yuan Y (2020). AvrA exerts inhibition of NF-κB pathway in its naïve *Salmonella* serotype through suppression of p-JNK and beclin-1 molecules. IJMS.

[201] Miller SI, Mekalanos JJ (1990). Constitutive expression of the phoP regulon attenuates *Salmonella* virulence and survival within macrophages. J Bacteriol.

[202] Verma S, Dixit R, Pandey KC (2016). Cysteine proteases: modes of activation and future prospects as pharmacological targets. Front Pharmacol.

[203] Kohler AC, Spanò S, Galán JE, Stebbins CE (2014). Structural and enzymatic characterization of a host-specificity determinant from *Salmonella*. Acta Crystallogr D Biol Crystallogr.

[204] Xu C, Kozlov G, Wong K, Gehring K, Cygler M (2016). Crystal structure of the *Salmonella* Typhimurium effector GtgE. PLoS One.

[205] Savitskiy S, Wachtel R, Pourjafar-Dehkordi D, Kang H-S, Trauschke V (2021). Proteolysis of Rab32 by *Salmonella* GtgE induces an inactive GTPase conformation. iScience.

[206] Wachtel R, Bräuning B, Mader SL, Ecker F, Kaila VRI (2018). The protease GtgE from *Salmonella* exclusively targets inactive Rab GTPases. Nat Commun.

[207] Spanò S, Gao X, Hannemann S, Lara-Tejero M, Galán JE (2016). A bacterial pathogen targets a host rab-family GTPase defense pathway with a GAP. Cell Host Microbe.

[208] Spanò S, Liu X, Galán JE (2011). Proteolytic targeting of Rab29 by an effector protein distinguishes the intracellular compartments of human-adapted and broad-host *Salmonella*. Proc Natl Acad Sci.

[209] Fu P, Zhang X, Jin M, Xu L, Wang C (2013). Complex structure of OspI and Ubc13: the molecular basis of Ubc13 deamidation and convergence of bacterial and host E2 recognition. PLoS Pathog.

[210] Russell AR, Ashfield T, Innes RW (2015). *Pseudomonas syringae* effector AvrPphB suppresses AvrB-induced activation of RPM1 but not AvrRpm1-induced activation. Mol Plant Microbe Interact.

[211] Grabe GJ, Zhang Y, Przydacz M, Rolhion N, Yang Y (2016). The *Salmonella* effector SpvD is a cysteine hydrolase with a serovar-specific polymorphism influencing catalytic activity, suppression of immune responses, and bacterial virulence. J Biol Chem.

[212] Rolhion N, Furniss RCD, Grabe G, Ryan A, Liu M (2016). Inhibition of nuclear transport of NF-ĸB p65 by the *Salmonella* type III secretion system effector SpvD. PLoS Pathog.

[213] McLaughlin LM, Govoni GR, Gerke C, Gopinath S, Peng K (2009). The *Salmonella* SPI2 effector SseI mediates long-term systemic infection by modulating host cell migration. PLoS Pathog.

[214] McLaughlin LM, Xu H, Carden SE, Fisher S, Reyes M (2014). A microfluidic-based genetic screen to identify microbial virulence factors that inhibit dendritic cell migration. Integr Biol.

[215] Bhaskaran SS, Stebbins CE (2012). Structure of the catalytic domain of the *Salmonella* virulence factor SseI. Acta Crystallogr D Biol Crystallogr.

[216] Brink T, Leiss V, Siegert P, Jehle D, Ebner JK (2018). *Salmonella* Typhimurium effector SseI inhibits chemotaxis and increases host cell survival by deamidation of heterotrimeric Gi proteins. PLoS Pathog.

[217] Hicks SW, Charron G, Hang HC, Galán JE (2011). Subcellular targeting of *Salmonella* virulence proteins by host-mediated S-palmitoylation. Cell Host Microbe.

[218] Carden SE, Walker GT, Honeycutt J, Lugo K, Pham T (2017). Pseudogenization of the secreted effector gene sseI confers rapid systemic dissemination of *S*. Typhimurium ST313 within migratory dendritic cells. Cell Host Microbe.

[219] Thornbrough JM, Worley MJ, Zhou D (2012). A naturally occurring single nucleotide polymorphism in the *Salmonella* SPI-2 type III effector srfH/sseI controls early extraintestinal dissemination. PLoS One.

[220] Baruch K, Gur-Arie L, Nadler C, Koby S, Yerushalmi G (2011). Metalloprotease type III effectors that specifically cleave JNK and NF-κB. EMBO J.

[221] Sun H, Kamanova J, Lara-Tejero M, Galán JE, Philpott DJ (2016). A family of *Salmonella* type III secretion effector proteins selectively targets the NF-κB signaling pathway to preserve host homeostasis. PLoS Pathog.

[222] Cerdà-Costa N, Gomis-Rüth FX (2014). Architecture and function of metallopeptidase catalytic domains. Protein Sci.

[223] Jennings E, Esposito D, Rittinger K, Thurston TLM (2018). Structure-function analyses of the bacterial zinc metalloprotease effector protein GtgA uncover key residues required for deactivating NF-κB. J Biol Chem.

[224] Giogha C, Lung TWF, Mühlen S, Pearson JS, Hartland EL (2015). Substrate recognition by the zinc metalloprotease effector NleC from enteropathogenic *Escherichia coli*. Cell Microbiol.

[225] Hodgson A, Wier EM, Fu K, Sun X, Yu H (2015). Metalloprotease NleC suppresses host NF-κB/inflammatory responses by cleaving p65 and interfering with the p65/RPS3 interaction. PLoS Pathog.

[226] Li W, Liu Y, Sheng X, Yin P, Hu F (2014). Structure and mechanism of a type III secretion protease, NleC. Acta Crystallogr D Biol Crystallogr.

[227] Pearson JS, Riedmaier P, Marchès O, Frankel G, Hartland EL (2011). A type III effector protease NleC from enteropathogenic *Escherichia coli* targets NF-κB for degradation. Mol Microbiol.

[228] Sham HP, Shames SR, Croxen MA, Ma C, Chan JM (2011). Attaching and effacing bacterial effector NleC suppresses epithelial inflammatory responses by inhibiting NF-κB and p38 mitogen-activated protein kinase activation. Infect Immun.

[229] Shames SR, Bhavsar AP, Croxen MA, Law RJ, Mak SHC (2011). The pathogenic *Escherichia coli* type III secreted protease NleC degrades the host acetyltransferase p300. Cell Microbiol.

[230] Stolle A-S, Norkowski S, Körner B, Schmitz J, Lüken L (2017). T3SS-independent uptake of the short-trip toxin-related recombinant NleC effector of enteropathogenic *Escherichia coli* leads to NF-κB p65 cleavage. Front Cell Infect Microbiol.

[231] Yen H, Ooka T, Iguchi A, Hayashi T, Sugimoto N (2010). NleC, a type III secretion protease, compromises NF-κB activation by targeting p65/RelA. PLoS Pathog.

[232] Viana F, Peringathara SS, Rizvi A, Schroeder GN (2021). Host manipulation by bacterial type III and type IV secretion system effector proteases. Cell Microbiol.

[233] Bos JL, Rehmann H, Wittinghofer A (2007). GEFs and GAPs: critical elements in the control of small G proteins. Cell.

[234] Cherfils J, Zeghouf M (2013). Regulation of small GTPases by GEFs, GAPs, and GDIs. Physiol Rev.

[235] Jiang X, Rossanese OW, Brown NF, Kujat-Choy S, Galán JE (2004). The related effector proteins SopD and SopD2 from *Salmonella enterica* serovar Typhimurium contribute to virulence during systemic infection of mice. Mol Microbiol.

[236] Bakowski MA, Cirulis JT, Brown NF, Finlay BB, Brumell JH (2007). SopD acts cooperatively with SopB during *Salmonella enterica* serovar Typhimurium invasion. Cell Microbiol.

[237] Boddy KC, Zhu H, D’Costa VM, Xu C, Beyrakhova K (2021). *Salmonella* effector SopD promotes plasma membrane scission by inhibiting Rab10. Nat Commun.

[238] Lian H, Jiang K, Tong M, Chen Z, Liu X (2021). The *Salmonella* effector protein SopD targets Rab8 to positively and negatively modulate the inflammatory response. Nat Microbiol.

[239] Savitskiy S, Itzen A (2021). SopD from *Salmonella* specifically inactivates Rab8. Biochim Biophys Acta Proteins Proteom.

[240] Luo L, Wall AA, Tong SJ, Hung Y, Xiao Z (2018). TLR crosstalk activates LRP1 to recruit Rab8a and PI3Kγ for suppression of inflammatory responses. Cell Reports.

[241] Tong SJ, Wall AA, Hung Y, Luo L, Stow JL (2021). Guanine nucleotide exchange factors activate Rab8a for Toll-like receptor signalling. Small GTPases.

[242] Wall AA, Luo L, Hung Y, Tong SJ, Condon ND (2017). Small GTPase Rab8a-recruited phosphatidylinositol 3-kinase γ regulates signaling and cytokine outputs from endosomal toll-like receptors. J Biol Chem.

[243] D’Costa VM, Braun V, Landekic M, Shi R, Proteau A (2015). *Salmonella* disrupts host endocytic trafficking by SopD2-mediated inhibition of Rab7. Cell Rep.

[244] Knuff-Janzen K, Serapio-Palacios A, McCoy J, Krekhno Z, Moon K-M (2021). Quantitative proteomic screen identifies annexin A2 as a host target for *Salmonella* pathogenicity island-2 effectors SopD2 and PipB2. Sci Rep.

[245] Schroeder N, Henry T, de Chastellier C, Zhao W, Guilhon A-A (2010). The virulence protein SopD2 regulates membrane dynamics of *Salmonella*-containing vacuoles. PLoS Pathog.

[246] Brumell JH, Kujat-Choy S, Brown NF, Vallance BA, Knodler LA (2003). SopD2 is a novel type III secreted effector of *Salmonella* typhimurium that targets late endocytic compartments upon delivery into host cells. Traffic.

[247] Teo WX, Yang Z, Kerr MC, Luo L, Guo Z (2017). *Salmonella* effector SopD2 interferes with Rab34 function. Cell Biol Int.

[248] Spanò S, Galán JE (2012). A Rab32-dependent pathway contributes to *Salmonella* Typhi host restriction. Science.

[249] Figueira R, Watson KG, Holden DW, Helaine S (2013). Identification of *Salmonella* pathogenicity island-2 type III secretion system effectors involved in intramacrophage replication of *S. enterica* serovar typhimurium: implications for rational vaccine design. mBio.

[250] Knuff-Janzen K, Tupin A, Yurist-Doutsch S, Rowland JL, Finlay BB (2020). Multiple *Salmonella*-pathogenicity island 2 effectors are required to facilitate bacterial establishment of its intracellular niche and virulence. PLoS One.

[251] Trombert AN, Rodas PI, Mora GC (2011). Reduced invasion to human epithelial cell lines of *Salmonella enterica* serovar Typhi carrying *S. Typhimurium* sopD2. FEMS Microbiol Lett.

[252] Stender S, Friebel A, Linder S, Rohde M, Mirold S (2000). Identification of SopE2 from *Salmonella* typhimurium, a conserved guanine nucleotide exchange factor for Cdc42 of the host cell. Mol Microbiol.

[253] Vonaesch P, Sellin ME, Cardini S, Singh V, Barthel M (2014). The *S almonella* Typhimurium effector protein SopE transiently localizes to the early SCV and contributes to intracellular replication. Cell Microbiol.

[254] Friebel A, Ilchmann H, Aepfelbacher M, Ehrbar K, Machleidt W (2001). SopE and SopE2 from *Salmonella* typhimurium activate different sets of RhoGTPases of the host cell. J Biol Chem.

[255] Buchwald G, Friebel A, Galán JE, Hardt W-D, Wittinghofer A (2002). Structural basis for the reversible activation of a Rho protein by the bacterial toxin SopE. EMBO J.

[256] Panagi I, Jennings E, Zeng J, Günster RA, Stones CD (2020). *Salmonella* effector SteE converts the mammalian serine/threonine kinase GSK3 into a tyrosine kinase to direct macrophage polarization. Cell Host Microbe.

[257] Gibbs KD, Washington EJ, Jaslow SL, Bourgeois JS, Foster MW (2020). The *Salmonella* secreted effector SarA/SteE mimics cytokine receptor signaling to activate STAT3. Cell Host Microbe.

[258] Jaslow SL, Gibbs KD, Fricke WF, Wang L, Pittman KJ (2018). *Salmonella* activation of STAT3 signaling by SarA effector promotes intracellular replication and production of IL-10. Cell Rep.

[259] Pham THM, Brewer SM, Thurston T, Massis LM, Honeycutt J (2020). *Salmonella*-driven polarization of granuloma macrophages antagonizes TNF-mediated pathogen restriction during persistent infection. Cell Host Microbe.

[260] Stapels DAC, Hill PWS, Westermann AJ, Fisher RA, Thurston TL (2018). *Salmonella* persisters undermine host immune defenses during antibiotic treatment. Science.

[261] Liu Z, Wang L, Yu Y, Fotin A, Wang Q (2022). SteE enhances the virulence of *Salmonella* pullorum in chickens by regulating the inflammation response. Front Vet Sci.

[262] Godlee C, Cerny O, Liu M, Blundell S, Gallagher AE (2022). The *Salmonella* transmembrane effector SteD hijacks AP1-mediated vesicular trafficking for delivery to antigen-loading MHCII compartments. PLoS Pathog.

[263] Bayer-Santos E, Durkin CH, Rigano LA, Kupz A, Alix E (2016). The *Salmonella* effector SteD mediates MARCH8-dependent ubiquitination of MHC II molecules and inhibits T cell activation. Cell Host Microbe.

[264] Alix E, Godlee C, Cerny O, Blundell S, Tocci R (2020). The tumour suppressor TMEM127 Is a Nedd4-family E3 ligase adaptor required by *Salmonella* SteD to ubiquitinate and degrade MHC class II molecules. Cell Host Microbe.

[265] Cerny O, Godlee C, Tocci R, Cross NE, Shi H (2021). CD97 stabilises the immunological synapse between dendritic cells and T cells and is targeted for degradation by the *Salmonella* effector SteD. PLoS Pathog.

[266] Pilar AVC, Reid-Yu SA, Cooper CA, Mulder DT, Coombes BK (2012). GogB Is an anti-inflammatory effector that limits tissue damage during *Salmonella* infection through interaction with human FBXO22 and Skp1. PLoS Pathog.

[267] Skowyra D, Craig KL, Tyers M, Elledge SJ, Harper JW (1997). F-Box proteins are receptors that recruit phosphorylated substrates to the SCF ubiquitin-ligase complex. Cell.

[268] Dai S, Zhou D (2004). Secretion and function of *Salmonella* SPI-2 effector SseF require its Chaperone, SscB. J Bacteriol.

[269] Deiwick J, Salcedo SP, Boucrot E, Gilliland SM, Henry T (2006). The translocated *Salmonella* effector proteins SseF and SseG interact and are required to establish an intracellular replication niche. Infect Immun.

[270] Müller P, Chikkaballi D, Hensel M, Webber MA (2012). Functional dissection of SseF, a membrane-integral effector protein of intracellular *Salmonella enterica*. PLOS One.

[271] Abrahams GL, Müller P, Hensel M (2006). Functional dissection of SseF, a type III effector protein involved in positioning the *Salmonella*-containing vacuole. Traffic.

[272] Salcedo SP, Holden DW (2003). SseG, a virulence protein that targets *Salmonella* to the Golgi network. EMBO J.

[273] Yu X-J, Liu M, Holden DW, Hultgren SJ (2016). *Salmonella* effectors SseF and SseG interact with mammalian protein ACBD3 (GCP60) to anchor *Salmonella*-containing vacuoles at the golgi network. mBio.

[274] Kuhle V, Hensel M (2002). SseF and SseG are translocated effectors of the type III secretion system of *Salmonella* pathogenicity island 2 that modulate aggregation of endosomal compartments. Cell Microbiol.

[275] Kuhle V, Jäckel D, Hensel M (2004). Effector proteins encoded by *Salmonella* pathogenicity island 2 interfere with the microtubule cytoskeleton after translocation into host cells. Traffic.

[276] Ramsden AE, Mota LJ, Münter S, Shorte SL, Holden DW (2007). The SPI-2 type III secretion system restricts motility of *Salmonella*-containing vacuoles. Cell Microbiol.

[277] Krieger V, Liebl D, Zhang Y, Rajashekar R, Chlanda P (2014). Reorganization of the endosomal system in Salmonella-infected cells: the ultrastructure of *Salmonella*-induced tubular compartments. PLoS Pathog.

[278] Moest T, Zhao W, Zhao Y, Schüssler JM, Yan W (2018). Contribution of bacterial effectors and host proteins to the composition and function of *Salmonella*-induced tubules. Cell Microbiol.

[279] Liss V, Swart AL, Kehl A, Hermanns N, Zhang Y (2017). *Salmonella enterica* remodels the host cell endosomal system for efficient intravacuolar nutrition. Cell Host Microbe.

[280] Noster J, Chao T-C, Sander N, Schulte M, Reuter T (2019). Proteomics of intracellular *Salmonella enterica* reveals roles of *Salmonella* pathogenicity island 2 in metabolism and antioxidant defense. PLoS Pathog.

[281] Kehl A, Göser V, Reuter T, Liss V, Franke M (2020). A trafficome-wide RNAi screen reveals deployment of early and late secretory host proteins and the entire late endo-/lysosomal vesicle fusion machinery by intracellular *Salmonella*. PLoS Pathog.

[282] Wang X, Li D, Qu D, Zhou D (2010). Involvement of TIP60 acetyltransferase in intracellular *Salmonella* replication. BMC Microbiol.

[283] Auweter SD, Bhavsar AP, de Hoog CL, Li Y, Chan YA (2011). Quantitative mass spectrometry catalogues *Salmonella* pathogenicity island-2 effectors and identifies their cognate host binding partners. J Biol Chem.

[284] Liss V, Hensel M (2015). Take the tube: remodelling of the endosomal system by intracellular *Salmonella enterica*. Cell Microbiol.

[285] McGourty K, Thurston TL, Matthews SA, Pinaud L, Mota LJ (2012). *Salmonella* inhibits retrograde trafficking of mannose-6-phosphate receptors and lysosome function. Science.

[286] Sindhwani A, Arya SB, Kaur H, Jagga D, Tuli A (2017). *Salmonella* exploits the host endolysosomal tethering factor HOPS complex to promote its intravacuolar replication. PLoS Pathog.

[287] Boucrot E, Henry T, Borg J-P, Gorvel J-P, Méresse S (2005). The intracellular fate of *Salmonella* depends on the recruitment of kinesin. Science.

[288] Kaniuk NA, Canadien V, Bagshaw RD, Bakowski M, Braun V (2011). *Salmonella* exploits Arl8B-directed kinesin activity to promote endosome tubulation and cell-to-cell transfer. Cell Microbiol.

[289] Rosa-Ferreira C, Munro S (2011). Arl8 and SKIP act together to link lysosomes to kinesin-1. Dev Cell.

[290] Henry T, Couillault C, Rockenfeller P, Boucrot E, Dumont A (2006). The *Salmonella* effector protein PipB2 is a linker for kinesin-1. Proc Natl Acad Sci.

[291] McEwan DG, Richter B, Claudi B, Wigge C, Wild P (2015). PLEKHM1 regulates *Salmonella*-containing vacuole biogenesis and infection. Cell Host Microbe.

[292] Jackson LK, Nawabi P, Hentea C, Roark EA, Haldar K (2008). The *Salmonella* virulence protein SifA is a G protein antagonist. Proc Natl Acad Sci.

[293] Arbeloa A, Garnett J, Lillington J, Bulgin RR, Berger CN (2010). EspM2 is a RhoA guanine nucleotide exchange factor. Cell Microbiol.

[294] Huang Z, Sutton SE, Wallenfang AJ, Orchard RC, Wu X (2009). Structural insights into host GTPase isoform selection by a family of bacterial GEF mimics. Nat Struct Mol Biol.

[295] Patel S, Wall DM, Castillo A, McCormick BA (2019). Caspase-3 cleavage of *Salmonella* type III secreted effector protein SifA is required for localization of functional domains and bacterial dissemination. Gut Microbes.

[296] Zhao W, Moest T, Zhao Y, Guilhon A-A, Buffat C (2015). The *Salmonella* effector protein SifA plays a dual role in virulence. Sci Rep.

[297] Reinicke AT, Hutchinson JL, Magee AI, Mastroeni P, Trowsdale J (2005). A *Salmonella* typhimurium effector protein SifA is modified by host cell prenylation and S-acylation machinery. J Biol Chem.

[298] Diacovich L, Dumont A, Lafitte D, Soprano E, Guilhon A-A (2009). Interaction between the SifA virulence factor and its host target SKIP is essential for *Salmonella* pathogenesis. J Biol Chem.

[299] Namakchian M, Kassler K, Sticht H, Hensel M, Deiwick J (2018). Structure-based functional analysis of effector protein SifA in living cells reveals motifs important for *Salmonella* intracellular proliferation. Int J Med Microbiol.

[300] McGhie EJ, Hayward RD, Koronakis V (2004). Control of actin turnover by a *Salmonella* invasion protein. Mol Cell.

[301] Singh PK, Kapoor A, Lomash RM, Kumar K, Kamerkar SC (2018). *Salmonella* SipA mimics a cognate SNARE for host Syntaxin8 to promote fusion with early endosomes. J Cell Biol.

[302] Auweter SD, Yu HB, Arena ET, Guttman JA, Finlay BB (2012). Oxysterol-binding protein (OSBP) enhances replication of intracellular Salmonella and binds the Salmonella SPI-2 effector SseL via its N-terminus. Microbes Infect.

[303] Arena ET, Auweter SD, Antunes LCM, Vogl AW, Han J (2011). The deubiquitinase activity of the *Salmonella* pathogenicity island 2 effector, SseL, prevents accumulation of cellular lipid droplets. Infect Immun.

[304] Young AM, Minson M, McQuate SE, Palmer AE (2017). Optimized fluorescence complementation platform for visualizing *Salmonella* effector proteins reveals distinctly different intracellular niches in different cell types. ACS Infect Dis.

[305] Meng K, Yang J, Xue J, Lv J, Zhu P (2022). A host E3 ubiquitin ligase regulates *Salmonella* virulence by targeting an SPI-2 effector involved in SIF biogenesis. Cell Biol.

[306] Geddes K, Worley M, Niemann G, Heffron F (2005). Identification of new secreted effectors in *Salmonella enterica* serovar Typhimurium. Infect Immun.

[307] Yeom J, Pontes MH, Choi J, Groisman EA (2018). A protein that controls the onset of a *Salmonella* virulence program. EMBO J.

[308] Cordero-Alba M, Bernal-Bayard J, Ramos-Morales F (2012). SrfJ, a *Salmonella* type III secretion system effector regulated by PhoP, RcsB, and IolR. J Bacteriol.

[309] Domingues L, Ismail A, Charro N, Rodríguez-Escudero I, Holden DW (2016). The *Salmonella* effector SteA binds phosphatidylinositol 4-phosphate for subcellular targeting within host cells. Cell Microbiol.

[310] McQuate SE, Young AM, Silva-Herzog E, Bunker E, Hernandez M (2017). Long-term live-cell imaging reveals new roles for *Salmonella* effector proteins SseG and SteA. Cell Microbiol.

[311] Gulati A, Shukla R, Mukhopadhaya A (2019). *Salmonella* effector SteA suppresses proinflammatory responses of the host by interfering with IκB degradation. Front Immunol.

[312] Lilic M, Vujanac M, Stebbins CE (2006). A common structural motif in the binding of virulence factors to bacterial secretion chaperones. Mol Cell.

[313] Barta ML, Dickenson NE, Patil M, Keightley A, Wyckoff GJ (2012). The structures of coiled-coil domains from type III secretion system translocators reveal homology to pore-forming toxins. J Mol Biol.

[314] Chatterjee S, Zhong D, Nordhues BA, Battaile KP, Lovell S (2011). The crystal structures of the *Salmonella* type III secretion system tip protein SipD in complex with deoxycholate and chenodeoxycholate. Protein Sci.

[315] Diao J, Zhang Y, Huibregtse JM, Zhou D, Chen J (2008). Crystal structure of SopA, a *Salmonella* effector protein mimicking a eukaryotic ubiquitin ligase. Nat Struct Mol Biol.

[316] Williams C, Galyov EE, Bagby S (2004). Solution structure, backbone dynamics, and interaction with Cdc42 of *Salmonella* guanine nucleotide exchange factor SopE2. Biochemistry.

[317] Stebbins CE, Galán JE (2001). Maintenance of an unfolded polypeptide by a cognate chaperone in bacterial type III secretion. Nature.

[318] Chen L, Wang H, Zhang J, Gu L, Huang N (2008). Structural basis for the catalytic mechanism of phosphothreonine lyase. Nat Struct Mol Biol.

[319] Kim Y-G, Kim J-H, Kim K-J (2009). Crystal structure of the *Salmonella enterica* serovar Typhimurium virulence factor Srfj, a glycoside hydrolase family enzyme. J Bacteriol.

[320] Wang L, Yan J, Niu H, Huang R, Wu S (2018). Autophagy and ubiquitination in *Salmonella* infection and the related inflammatory responses. Front Cell Infect Microbiol.

[321] Zhang W, Sidhu SS (2014). Development of inhibitors in the ubiquitination cascade. FEBS Lett.

[322] Horn-Ghetko D, Schulman BA (2022). New classes of E3 ligases illuminated by chemical probes. Curr Opin Struct Biol.

[323] Oh E, Akopian D, Rape M (2018). Principles of ubiquitin-dependent signaling. Annu Rev Cell Dev Biol.

[324] Kocaturk NM, Gozuacik D (2018). Crosstalk between mammalian autophagy and the ubiquitin-proteasome system. Front Cell Dev Biol.

[325] Thurston TLM, Ryzhakov G, Bloor S, von Muhlinen N, Randow F (2009). The TBK1 adaptor and autophagy receptor NDP52 restricts the proliferation of ubiquitin-coated bacteria. Nat Immunol.

[326] Wild P, Farhan H, McEwan DG, Wagner S, Rogov VV (2011). Phosphorylation of the autophagy receptor optineurin restricts *Salmonella* growth. Science.

[327] Ciechanover A, Schwartz AL (1998). The ubiquitin-proteasome pathway: the complexity and myriad functions of proteins death. Proc Natl Acad Sci.

[328] Coux O, Tanaka K, Goldberg AL (1996). Structure and functions of the 20s and 26s proteasomes. Annu Rev Biochem.

[329] Mani A, Gelmann EP (2005). The ubiquitin-proteasome pathway and its role in cancer. J Clin Oncol.

